# Genetic landscape of extreme responders with anaplastic oligodendroglioma

**DOI:** 10.18632/oncotarget.16773

**Published:** 2017-03-31

**Authors:** Matthias Holdhoff, Gregory J. Cairncross, Thomas M. Kollmeyer, Ming Zhang, Peixin Zhang, Minesh P. Mehta, Maria Werner-Wasik, Luis Souhami, Jean-Paul Bahary, Young Kwok, Alan C. Hartford, Arnab Chakravarti, Srinivasan Yegnasubramanian, Bert Vogelstein, Nickolas Papadopoulos, Kenneth Kinzler, Robert B. Jenkins, Chetan Bettegowda

**Affiliations:** ^1^ The Sidney Kimmel Comprehensive Cancer Center at Johns Hopkins, Baltimore, MD, USA; ^2^ Charbonneau Cancer Institute at the University of Calgary, Calgary, AB, USA; ^3^ Mayo Clinic, Rochester, MN, USA; ^4^ NRG Oncology Statistics and Data Management Center, Philadelphia, PA, USA; ^5^ University of Maryland Medical Center, Baltimore, MD, USA; ^6^ Thomas Jefferson University Hospital, Philadelphia, PA, USA; ^7^ McGill University Health Centre, Montreal, QC, Canada; ^8^ Centre Hospitalier de l'Université de Montréal, Montreal University, Montreal, QC, Canada; ^9^ Darthmouth-Hitchcock Medical Center, Lebanon, NH, USA; ^10^ The Ohio State University, Columbus, OH, USA

**Keywords:** oligodendroglioma, chemotherapy, survival, genomics, co-deletion 1p/19q

## Abstract

**Background:**

The NRG Oncology RTOG 9402 trial showed significant survival benefit in patients with 1p/19q co-deleted anaplastic oligodendrogliomas (AO) who received both radiation (RT) and chemotherapy (PCV regimen) versus RT alone. Substantial separation of the survival curves was only seen after 7.3 years. We aimed to determine whether there are specific genetic alterations that distinguish co-deleted AO patients who benefit from the addition of PCV from those who do not. Methods: We performed whole exome sequencing on matched tumor and normal DNA from all available short-term (STS) and long-term survivors (LTS) who received RT+PCV. *hTERT* status and rs55705857 genotypes (G-allele) were analyzed in both cohorts. Results: Six STS (survival of <7.3y) and 7 LTS (survival of ≥7.3y and no progression) had sufficient material for analysis. There was no significant difference between the groups regarding age, performance status and extent of resection. On average, STS had 7 and LTS 4 mutations. Most common mutations in STS *vs*. LTS were: *IDH1* (67 vs. 86%), *CIC* (50 *vs*. 71%) and *FUBP1* (17 *vs*. 71%). The *hTERT* promoter was mutated in 83% STS and 86% LTS. Genotyping of rs55705857 showed a higher prevalence of G allele carriers in LTS than STS (43 *vs*. 17%).

**Conclusions:**

These findings confirm that *IDH*, *CIC*, *FUBP1* mutations and rs55705857 genotype are common in AO. No distinct genetic signature was identified to differentiate STS and LTS.

## INTRODUCTION

Oligodendrogliomas are the second most common adult primary brain tumor, constituting about 20% of all glial tumors. They typically present in the fourth to sixth decade of life and are classified by the World Health Organization as grade II (low grade) or grade III (anaplastic oligodendroglioma, AO). For a number of years, it has been known that the presence of a co-deletion of the short arm of chromosome 1 and the long arm of chromosome 19 (1p/19q co-deletion), arising from an unbalanced pericentromeric translocation, is associated with significantly improved survival as well as response to chemotherapy in patients with oligodendrogliomas [1, 2].

Our understanding of the molecular basis for oligodendroglioma formation has rapidly advanced in recent years. In addition to 1p/19q co-deletion, it has become well recognized that mutations in IDH1/2 are present in the vast majority of oligodendrogliomas [5, 6]. More recent studies have implicated inactivation of *CIC* and *FUBP1* as central mechanisms for oligodendroglioma pathogenesis [6–8]. Mutations in the *TERT* promoter are present in ~80% of oligodendrogliomas and are mutually exclusive of ATRX mutations commonly seen in non-oligodendroglial lower grade gliomas [6, 9]. Furthermore, alterations in *NOTCH1* and *PIK3CA* are commonly found in oligodendroglial neoplasms [6, 7]. The molecular characteristics of lower grade gliomas, including oligodendroglioma, have become refined to a level that is permitting molecular classification schemes based on genomic alterations and not histological findings. For example, 1p/19q co-deletion, *IDH1/2* and *TERT* promoter mutation are oligodendroglioma defining alterations [10, 11].

**Figure 1:**
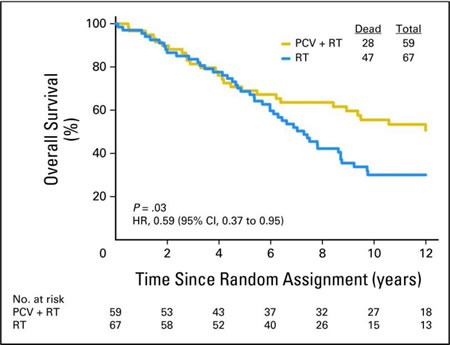
Patients with 1p and 19q co-deleted anaplastic oligodendrogliomas treated with radiation (blue curve) versus PCV and radiation (gold curve) within NRG Oncology RTOG 9402 [1] Reprinted with permission. ©2013 American Society of Clinical Oncology. Cairncross JG et al: Phase III trial of chemoradiotherapy for anaplastic oligodendrogliomas: long-term results of RTOG 9402. J Clin Oncol 2013;31:337-343.

Recently, two landmark studies, the Radiation Therapy Oncology Group (RTOG) study NRG Oncology RTOG 9402 and the European study, EORTC 26951, identified a significant long-term benefit in overall survival in patients with co-deleted AO if chemotherapy with the PCV regimen (procarbazine, lomustine, vincristine) was added to radiation [3, 12]. The two studies were independently conducted and showed virtually identical results. NRG Oncology RTOG 9402 randomized patients with AO to 4 cycles of PCV with a lomustine dose of 130 mg/m^2^ followed by radiation, versus radiation alone. Patients in the EORTC 26951 trial received radiation followed by up to 6 cycles of PCV (lomustine dose 110 mg/m^2^) in the combination therapy arm. Both studies demonstrated that there was no inter-arm difference in overall survival between both the combination therapy and the radiation monotherapy group for approximately the first 7 years of the trial [1, 2, 4, 13]. However, the curves started to separate thereafter [3, 12]. Impressive differences in overall survival were found upon long-term outcome analysis of both trials. NRG Oncology RTOG 9402 showed a median overall survival of 7.3 versus 14.7 years in patients who received RT alone versus patients who received combination therapy with PCV plus RT. Similarly, median survival in EORTC 26951 was 9.3 years in the RT arm and median survival in the combination therapy arm had not been reached at time of publication. The results of the two studies show a subgroup of patients who clearly derive benefit from the addition of PCV to RT (i.e. patients who were at the tail end of the combination therapy curve), whereas other patients did not appear to benefit from the addition of PCV chemotherapy (i.e. patients who died prior to the 7-year mark, Figure 1). The reason for this difference is still unknown.

Our aim was to determine the genetic landscape of extreme responders in NRG Oncology RTOG 9402 and to identify alterations that could be predictive of benefit from PCV. Study NRG Oncology RTOG 9402 was suitable for this type of analysis as both tumor tissue as well as normal DNA had been collected prospectively, which is a prerequisite for analysis of somatic alterations.

## RESULTS

### Patient cohorts and demographic information

A total of 59 patients with co-deleted AO were part of the combination therapy arm in NRG Oncology RTOG 9402. All extreme short- and long-term survivors for whom sufficient tissue was available for analysis were included in this study. “Short-term survivors” (poor outcome patients; STS) were defined as patients who had died of their disease prior to 7.3 years on trial. “Long-term survivors” (good outcome patients, extreme responders; terms used synonymously in this manuscript; LTS) were patients who had never progressed based on available follow-up data.

After comprehensive analysis of clinical outcome data and tissue availability in the RTOG biospecimen bank of the 59 1p/19q co-deleted AO patients on the combination treatment arm, we were able to identify FFPE tumor and normal samples derived from 6 STS and 7 LTS that were suitable for mutational analysis. For three additional samples that underwent quality control (two in the STS and one in the LTS cohort), DNA quality was too poor to perform whole exome sequencing. Because of the small number of evaluable specimens, especially in the STS cohort, we then searched for potential patients as STS who had lived beyond 7.3 years, but who had clear disease progression. We identified two patients, one with progression after 2.5 years, but who survived for 9.5 years, and another one who progressed after 1.6 years, but who lived to 14.6 years. These two patients were analyzed with whole exome sequencing the same way as the other samples, but they were not included in our comparative analysis (STS vs. LTS) due to their long overall survival. Sequencing results for samples of these two patients are included in Table 2 (‘Added patient 1′ and ‘Added patient 2′). The final number of patients in the STS versus the LTS group that were successfully sequenced was 6 and 7, respectively (n=13).

**Table 1:** Patient pretreatment characteristics

	Short-term Survivors (*n*=6)	Long-term Survivors (*n*=7)

Age* (years)		
Median	56.5	46
Min - Max	43 - 65	32 - 52
Q1 - Q3	44 - 62	34 - 51
<50	2 (33.3%)	4 (57.1%)
50+	4 (66.7%)	3 (42.9%)
Gender		
Male	5 (83.3%)	3 (42.9%)
Female	1 (16.7%)	4 (57.1%)
Race		
White	5 (83.3%)	5 (71.4%)
Oriental	1 (16.7%)	1 (14.3%)
Other	0 (0.0%)	1 (14.3%)
Karnofsky performance Status*		
60-70	0 (0.0%)	1 (14.3%)
80-100	6 (100.0%)	6 (85.7%)
Prior surgery		
Biopsy	0 (0.0%)	1 (14.3%)
Partial Resection	3 (50.0%)	5 (71.4%)
Total Resection	3 (50.0%)	1 (14.3%)
Neurological function		
No symptoms	1 (16.7%)	2 (28.6%)
Minor symptoms	4 (66.7%)	3 (42.9%)
Moderate (fully active)	0 (0.0%)	2 (28.6%)
Moderate (not fully active)	1 (16.7%)	0 (0.0%)
Histology		
Anaplastic oligodendroglioma	5 (83.3%)	6 (85.7%)
Anaplastic oligoastrocytoma, oligo dominant	1 (16.7%)	1 (14.3%)
Grade*		
Moderatly Anaplastic	3 (50.0%)	6 (85.7%)
Very Anaplastic	3 (50.0%)	1 (14.3%)

* stratification factor Ql = first quartile; Q3 = third quartile.

**Table 2:** Exome mutations, *hTERT* promoter mutations and G-allele status of anaplastic oligodendroglioma patients with short-term *versus* long-term survival treated with RT and PCV within NRG Oncology RTOG 9402

	ST-1	ST-2	ST-3	ST-4	ST-5	ST-6	LT-1	LT-2	LT-3	LT-4	LT-5	LT-6	LT-7	Added patient 1	Added patient 2	Total number mutated
IDH1	-	M	-	M	M	M	M	M	M	M	M	M	-	M	-	11
CIC	-	M	M	-	M	-	M	-	M	M	M	M	-	-	-	8
FUBP1	-	-	-	-	M	-	-	M	M	M	M	-	M	M	M	8
NOTCH1	-	-	M	-	-	-	-	M	-	-	-	-	-	-	M	3
NIPBL	M	-	M	-	-	-	-	M	-	-	-	-	-	-	-	3
ESX1	-	-	M	-	-	-	-	-	-	-	M	M	-	-	-	2
PIK3CA	-	M	-	-	M	-	-	-	M	-	-	-	-	-	-	3
AC005014.2	-	-	M	-	-	M	-	-	-	-	-	-	-	-	-	2
EIF3L	-	-	M	M	-	-	-	-	-	-	-	-	-	-	-	2
FAM83A	-	-	M	-	-	-	-	-	-	M	-	-	-	-	-	2
RIF1	-	M	-	-	-	-	-	-	-	-	-	M	-	-	-	2
RLBP1L2	-	-	M	M	-	-	-	-	-	-	-	-	-	-	-	2
TTN	-	M	-	-	-	-	M	-	-	-	-	-	-	-	-	2
EIF4B	-	-	-	-	-	-	-	-	-	-	-	-	-	-	M	1
EIF4E3	-	-	-	-	M	-	-	-	-	-	-	-	-	-	-	1
EPC2	-	-	-	-	M	-	-	-	-	-	-	-	-	-	-	1
KDM6B	-	-	-	-	M	-	-	-	-	-	-	-	-	-	-	1
ATRX	-	-	-	-	-	-	-	-	-	-	-	M	-	-	-	1
NOTCH2	-	-	-	M	-	-	-	-	-	-	-	-	-	-	-	1
NOTCH3	-	-	-	-	-	-	-	M	-	-	-	-	-	-	-	1
HOXD3	-	-	-	-	-	-	-	-	M	-	-	-	-	-	-	1
EEF1G	-	-	M	-	-	-	-	-	-	-	-	-	-	-	-	1
EIF3L	-	-	M	-	-	-	-	-	-	-	-	-	-	-	-	1
EPHA7	-	-	M	-	-	-	-	-	-	-	-	-	-	-	-	1
HDAC1	-	-	M	-	-	-	-	-	-	-	-	-	-	-	-	1
MGMT	-	-	M	-	-	-	-	-	-	-	-	-	-	-	-	1
MLL	-	-	M	-	-	-	-	-	-	-	-	-	-	-	-	1
MLL2	-	-	M	-	-	-	-	-	-	-	-	-	-	-	-	1
PIK3AP1	-	-	M	-	-	-	-	-	-	-	-	-	-	-	-	1
TP53	-	-	M	-	-	-	-	-	-	-	-	-	-	-	-	1
HDAC2	-	-	-	-	-	-	M	-	-	-	-	-	-	-	-	1
G-Allele	AA	AA	AA	AA	GA	AA	GA	AA	AA	GA	AA	AA	GA	GA	AA	N/A
hTERT	-	M	M	M	M	M	M	M	M	M	M	M	-	M	M	
Survival (years)	0.5	0.5	4.2	4.9	5.5	6.4	8	11.2	11.3	12	13.3	16.2	16.8	9.5	14.6	
PFS (years)	0.2	0.5	0.9	4.9	0.7	1.9	8	11.2	11.3	12	13.3	16.2	16.8	2.5	1.6	
Progressive disease?	Yes	N/A*	Yes	N/A*	Yes	Yes	No	No	No	No	No	No	No	Yes	Yes	

Survival data for the individual patients are shown. PFS = Progression free survival. *Death, unrelated to disease progression.

Baseline demographic information and known prognostic factors of the 13 patients, including age at diagnosis, Karnofsky performance status (KPS), and extent of resection, are summarized in Table 1. Patient pretreatment characteristics between the two groups did not explain the survival difference between the two groups of patients. The numbers were too small to be tested for statistical significance. Pretreatment characteristics of the 15 analyzed patients (i.e., 6 STS, 7 LTS and the 2 added patients who had progressed but who survived beyond 7.3 years) were not significantly different from those of the remaining 44 patients with 1p/19q co-deletion who had received RT plus PCV in NRG Oncology RTOG 9402 (Supplementary Table 1). There was no difference in overall survival between these two groups of patients. Similarly, no statistically significant difference in patient pretreatment characteristics was observed between these 15 patients analyzed in this study and the remaining 276 patients that were part of RTOG 9402 (Supplementary Table 2).

### Germline and mutational analysis

We performed whole exome sequencing on matched normal and tumor DNA for both STS and LTS cohorts (n=13) as well as for two additional samples. The average distinct high quality coverage of the tumors was similar between the two groups, with 68 ± 44 (SD) distinct reads for STS and 104 ± 46 or LTS.

A total of 31 different genes were found to be mutated in the total of 15 sequenced samples. The most frequently mutated gene was *IDH1* (mutated in 11 of 15 patients; 67% STS and 86% LTS), followed by *CIC* (mutant in 8 of 15 patients; 50% in STS and 71% in LTS) and *FUBP1* (mutant in 8 of 15 patients; 17% STS and 71% LTS). The mutational landscape of the analyzed patients is shown in Table 2. Due to its known relevance in oligodendrogliomas, we also analyzed mutations within the *hTERT* promoter. *hTERT* promoter mutations were detected in 83% of STS and in 86% of LTS [9].

In addition, we determined the genotype of rs55705857 as this had recently been described as a relevant molecular marker for this disease with prognostic implication in *IDH1* mutated patients [14]. Genotype rs55705857 was detected in 17% STS and in 43% LTS (Table 2).

In summary, mutations in *IDH1*, *CIC*, *FUBP1* and *TERT* promoter mutations were more frequently detected in LTS versus STS, although these differences were not statistically significant due to the small sample size. Similarly, genotype rs55705857 was more common in LTS than in STS. The only other recurrently mutated genes (detected in ≥ 3 patients when combining both groups) were *NIPBL1, PIK3CA* and *ESX1* [6, 7]. *NIPBL* is a regulatory subunit of the cohesin complex that plays a central role in chromatin structure that has been implicated in other cancers but not oligodendroglioma [15]. *ESX1* had been previously reported to be a driver of neoplastic processes involving lower grade gliomas [16].

A comprehensive list of all exome sequencing results of this study (all mutations of all patients analyzed) can be found in Supplementary Table 3.

## DISCUSSION

Understanding why some patients respond to specific therapies while others do not remains a central question for most tumor types. NRG Oncology RTOG 9402 presented a unique opportunity to further study extreme responders and identify possible genetic determinants of response. Certain key criteria must be met in order to perform detailed genomic analysis in patients with disparate clinical results. The first must be an extremely well curated database of patients, ideally treated in a prospective, codified manner. Second, the results from the clinical trial should clearly demonstrate that a fraction of patients derive significant survival benefit from the therapy, while another fraction does not. Third, there should be no confounding demographic, clinical or known prognostic factor that could explain the difference in outcomes. Fourth, the requisite biological material, in our case DNA derived from tumor and normal cells, must be available. Lastly, it is crucial to have sufficient number of patient derived samples to be able to discern differences between distinct clinical groups. A significant limitation in the current study is the lack of a large repository of high quality tissue samples that could be analyzed to identify and validate genomic differences between STS and LTS.

Despite this limitation, because AO are rare neoplasms and NRG Oncology RTOG 9402 is one of the seminal studies that formed the basis for the current treatment paradigm, we explored the genetic basis of STS and LTS. There are a few pertinent insights from this exploratory analysis: First, the study did not identify a distinct genetic signature in STS versus LTS with co-deleted AO. A primary reason for this could be the lack of available tissue in both groups to perform a highly powered analysis. Second, our study highlighted challenges in using historical FFPE tissue samples (from 1994-2002), even if these had been prospectively collected and adequately stored. They were received after patient enrollment on this trial and stored in the dark at room temperature. Third, the results of our analysis confirmed the presence of a number of previously described somatic mutations in AO, namely mutations of *IDH1*, *CIC* and *FUBP1* [5–8, 17]. The most commonly detected somatic mutation was *IDH1*, which was mutated in 4 of 6 (67%) of STS and 6 of 7 (86%) in LTS. In addition, mutations of *CIC* and *FUBP1*, and also the G-allele (genotype rs55705857) were found more frequently in LTS versus STS. However, the limited number of cases did not allow for calculation of statistical significance of any of these markers.

*IDH* mutations are now also part of the new WHO classification of oligodendrogliomas and anaplastic oligodendrogliomas [18]. Interestingly, there was absence of an IDH mutation in a total of 4 cases of this analysis, raising the question of whether the tumors were truly 1p/19q co-deleted or not. At time the clinical trial was conducted, FISH analysis was used as the standard testing method for co-deletions of 1p and 19q. Since then, we have learned that more comprehensive methods such as SNP array studies are superior to FISH as they definitively answer the question of whether an entire deletion of 1p and 19q is present or not. This is of clinical relevance as tumors that are not truly co-deleted, although possibly FISH positive, would not typically behave like anaplastic oligodendrogliomas, and would be better classified as high-grade astrocytomas [11, 18, 19].

A number of additional mutations were found in individual patients, but there was no clear pattern of distribution between groups and the significance of these is unclear. Of note is patient ST-3, a short-term survivor whose tumor harbored far more mutations (17 compared to the observed mean of 7) than any of the other samples analyzed. The significance of this is also unclear. In addition to whole exome analysis, we chose to also selectively include G-allele (genotype rs55705857) testing, as this had recently been described as a relevant molecular marker for this disease with prognostic implication in *IDH1* mutated patients [14]. G-allele was indeed more frequently detected in LTS compared to STS (detected in 3 of 7 compared to only 1 of 6 patients, respectively). Although noteworthy, the same limitation of small sample size also applies here.

Taken together, there was no clearly distinct genomic or mutational signature that exclusively separated the two groups. The reason for the distinct survival difference cannot be readily explained by mutations in the coding region of the genome, *hTERT* mutations or G-allele status. Other molecular mechanisms may play a role but these are yet to be determined.

Our study has several limitations. First, our analysis was restricted to a small number of patients, despite screening all individuals enrolled in NRG Oncology RTOG 9402 for eligibility. The major limiting factor was the lack of sufficient matched normal and tumor DNA from patients enrolled in the study. This was in part due to the fact that there were only 59 co-deleted AO patients in the combination therapy arm of NRG Oncology RTOG 9402. In addition, tissue samples were insufficient in quantity or quality in a number of patients, which further reduced the number of patients that could be included in this analysis. It is also possible that the DNA quality obtained from ~ 15-20 year old FFPE blocks was not sufficient to permit uniform amplification and sequencing, which could lead to under-representation of somatic alterations within the tumor samples. Nonetheless, baseline demographic and prognostic characteristics were similar between the two studied cohorts and were representative of the characteristics of the remaining subjects that were enrolled in NRG Oncology RTOG 9402. The study was performed using the only available prospectively collected tissue collection of both tumor and paired normal DNA in a study comparing a therapeutic intervention (PCV added to RT) in patients with co-deleted AO. Therefore, the available data are to date the only comprehensive genomic analysis for somatic mutations in these patients to address the question of chemosensitivity in patients with co-deleted AO. A similar or improved study can be performed if tumor and matched normal DNA is collected in appropriate fashion in future prospective studies involving these tumors. Completion of such a study however takes many years due to the relatively low incidence and relatively good prognosis of AO compared to other gliomas.

Genomic analysis of tumors has become commonplace over the past five years and with technological advances, the results are available faster, tissue requirements have diminished and cost has dramatically decreased. In order to avoid challenges with archived FFPE tissues, one may consider banking appropriate biospecimens for future analysis or incorporate comprehensive genomic analysis as a pre-planned component of future clinical trials. Such analysis could be routinely incorporated into the interpretation of outcomes of future trials and hopefully provide additional insights into which patients may be most likely to derive clinical benefit.

## MATERIALS AND METHODS

This is a retrospective analysis of prospectively collected specimens from NRG Oncology RTOG 9402. The work was done with formal approvals by the NRG Oncology RTOG Translational Research Program (TRP) and by the respective Institutional Review Boards of the participating institutions.

### Sample acquisition and storage

Patient enrollment and sample acquisition has been described previously [17]. Tumor DNA was derived from FFPE samples. FFPE tissue and blood tubes were sent to the Biospecimens Accessioning and Processing Core at the Mayo Clinic. Buffy coats were isolated from blood samples and were used to produce both germline DNA and EBV transformed leukocytes. Prior to DNA extraction, buffy coats and EBV transformed leukocytes were stored at -80°C. FFPE blocks and slides were stored under ambient conditions. DNA was frozen at -20°C for short-term storage and -80°C for long-term storage.

### DNA extraction

Germline DNA was obtained from buffy coats or Epstein-Barr virus-transformed leukocytes using the DNeasy Blood and Tissue kit (Qiagen, Valencia, CA). Tumor DNA was extracted from macrodissected 5 or 10 micron FFPE tissue sections using the QIAamp DNA FFPE Tissue Kit (Qiagen). Area to be macrodissected was identified by neuropathology review of parallel section stained with hematoxylin and eosin. Both germline and tumor DNA concentrations were determined using the Qubit dsDNA BR Assay Kit (Invitrogen/Life Technologies, Grand Island, NY).

### hTERT sequencing

Sequences of the TERT promoter region were obtained from the human reference sequence (GRCh37/hg19; http://genome.ucsc.edu/) and amplified by PCR. Primers with the sequences 5′-M13-GTC CTG CCC CTT CAC CTT C-3′ and 5′-CAG CGC TGC CTG AAA CTC-3′, where M13 is a universal sequencing priming site with sequence 5′-tgtaaaacgacggccagt-3′, were used to amplify a 163-bp product containing C228T and C250T hotspot mutations in the TERT promoter region [19]. PCR amplification of DNA from frozen tissue samples was performed in a 25ul solution, consisting of 3ng of DNA solution, 0.1 μl of Phusion High-Fidelity DNA Polymerase (2 U/μl), 5 μl of 5X HF Buffer, 0.5 μl of dNTP mix(10 mM each), 10% (v/v) DMSO, and 1.25 μl of each primer (10 μM). PCR was conducted using a PTC-200 thermal cycler (BIO-RAD, CA, USA) with an initial denaturation step at 95°C for 2 min, followed by 3 cycles of denaturation at 95°C for 20 s, annealing at 64°C for 15 s, extension at 72°C for 5 s, and 3 cycles of denaturation at 95°C for 20 s, annealing at 61°C for 15 s, extension at 72°C for 5 s, and 3 cycles of denaturation at 95°C for 20 s, annealing at 58°C for 15 s, and 35 cycles of denaturation at 95°C for 20 s, annealing at 57°C for 15 s, extension at 72°C for 5 s, and a final extension at 72°C for 10 min. The amplified products were sent to GeneWIZ (MD, USA) for Sanger sequencing. Mutation status was determined using Mutation Surveyor (SoftGenetics, USA).

### G-Allele sequencing analysis

G (*v* A) allele of rs55705857 is a germ-line polymorphism associated with a six-fold increased risk of developing *IDH*-mutated glioma. Genotyping of germline DNA was performed as previously described [14, 17].

### Whole exome sequencing

DNA from matched tumor and normal tissue was used to generate libraries suitable for next generation sequencing as previously described [21–23]. DNA libraries were sequenced using the Illumina HiSeq and MiSeq genome analyzers.

### Bioinformatics analysis

Bioinformatic analyses were performed at Personal Genome Diagnostics (Baltimore, MD) as previously described [23]. Somatic mutations were identified using VariantDx custom software for identifying mutations in matched tumor and normal samples. Prior to mutation calling, primary processing of sequence data for both tumor and normal samples were performed using Illumina CASAVA software (v1.8), including masking of adapter sequences. Sequence reads were aligned against the human reference genome (version hg18) using ELAND. Candidate somatic mutations, consisting of point mutations, insertions, and deletions were then identified using VariantDx across either the whole exome, or regions of interest. VariantDx examines sequence alignments of tumor samples against a matched normal while applying filters to exclude alignment and sequencing artifacts. In brief, an alignment filter was applied to exclude quality failed reads, unpaired reads, and poorly mapped reads in the tumor. A base quality filter was applied to limit inclusion of bases to those with reported phred quality score > 30 for the tumor and > 20 for the normal. A mutation in the tumor was identified as a candidate somatic mutation only when (i) distinct paired reads contained the mutation in the tumor; (ii) the number of distinct paired reads containing a particular mutation in the tumor was at least 2% of the total distinct read pairs for targeted analyses and 10% of read pairs for exome and (iii) the mismatched base was not present in >1% of the reads in the matched normal sample as well as not present in a custom database of common germline variants derived from dbSNP and (iv) the position was covered in both the tumor and normal. Mutations arising from misplaced genome alignments, including paralogous sequences, were identified and excluded by searching the reference genome.

Candidate somatic mutations were further filtered based on gene annotation to identify those occurring in protein coding regions. Functional consequences were predicted using snpEff and a custom database of CCDS, RefSeq and Ensembl annotations using the latest transcript versions available on hg18 from UCSC (https://genome.ucsc.edu/). Predictions were ordered to prefer transcripts with canonical start and stop codons and CCDS or Refseq transcripts over Ensembl when available. Finally, mutations were filtered to exclude intronic and silent changes, while retaining mutations resulting in missense mutations, nonsense mutations, frameshifts, or splice site alterations. A manual visual inspection step was used to further remove artefactual changes. Similar approaches have been used in previous studies to successfully identify somatic mutations with high fidelity [22–23].

### Statistical analysis

Given the limited number of patients with biomarker information, descriptive statistics were mainly used to summarize the findings of this study. Mutation status for each biomarker and patient baseline characteristics were reported by survival status (STS vs. LTS). Missing data analyses were performed by comparing patient baseline characteristics and survival status between patients with and without biomarker information for all the patients with 1p/19q co-deletion and treated with RT plus PCV. Patient baseline characteristics were also compared between the patients involved in this study and the rest of eligible patients in NRG Oncology RTOG 9402. Chi-square tests were used to test statistical significance.

## SUPPLEMENTARY TABLES






