# Supplementary material for: Genetic landscape of extreme responders with anaplastic oligodendroglioma
Source: Oncotarget. 2017 Mar 31;8(22):35523–31. doi: 10.18632/oncotarget.16773 (PMC5482595; doi:10.18632/oncotarget.16773)
Supplement: Supplementary file 1 [file oncotarget-08-35523-s001.docx]

| **Supplementary Table 1.** Pretreatment characteristics of patients in this tissue repository study (TRP206) compared to the remaining 1p/19q co-deleted patients on the RT + PCV arm in RTOG 9402 | | | |
| --- | --- | --- | --- |
|  | In TRP 206 (n=15) | Not in TRP 206 (n=44) | Chi-square  p-value |
|  | | | |
| Age* (years) |  |  | 0.23 |
| <50 | 8 ( 53.3%) | 31 ( 70.5%) |  |
| 50+ | 7 ( 46.7%) | 13 ( 29.5%) |  |
|  | | | |
| Gender |  |  | 0.95 |
| Male | 9 ( 60.0%) | 26 ( 59.1%) |  |
| Female | 6 ( 40.0%) | 18 ( 40.9%) |  |
|  | | | |
| Race |  |  | 0.08 |
| White | 12 ( 80.0%) | 40 ( 90.9%) |  |
| Hispanic | 0 ( 0.0%) | 3 ( 6.8%) |  |
| Oriental | 2 ( 13.3%) | 1 ( 2.3%) |  |
| Other | 1 ( 6.7%) | 0 ( 0.0%) |  |
|  | | | |
| Karnofsky performance Status* |  |  | 0.77 |
| 60-70 | 1 ( 6.7%) | 4 ( 9.1%) |  |
| 80-100 | 14 ( 93.3%) | 40 ( 90.9%) |  |
|  | | | |
| Prior surgery |  |  | 0.95 |
| Biopsy | 1 ( 6.7%) | 4 ( 9.1%) |  |
| Partial Resection | 9 ( 60.0%) | 25 ( 56.8%) |  |
| Total Resection | 5 ( 33.3%) | 15 ( 34.1%) |  |
|  | | | |
| Neurological function |  |  | 0.88 |
| No symptoms | 5 ( 33.3%) | 15 ( 34.1%) |  |
| Minor symptoms | 7 ( 46.7%) | 21 ( 47.7%) |  |
| Moderate (fully active) | 2 ( 13.3%) | 7 ( 15.9%) |  |
| Moderate (not fully active) | 1 ( 6.7%) | 1 ( 2.3%) |  |
|  | | | |
| Histology |  |  | 0.42 |
| Anaplastic oligodendroglioma | 12 ( 80.0%) | 35 ( 79.5%) |  |
| Anaplastic oligoastrocytoma, oligo dominant | 3 ( 20.0%) | 4 ( 9.1%) |  |
| Anaplastic oligoastrocytoma, oligo=astro | 0 ( 0.0%) | 3 ( 6.8%) |  |
| Anaplastic oligoastrocytoma, astro dominant | 0 ( 0.0%) | 2 ( 4.5%) |  |
|  | | | |
| Grade* |  |  | 0.26 |
| Moderatly Anaplastic | 10 ( 66.7%) | 22 ( 50.0%) |  |
| Very Anaplastic | 5 ( 33.3%) | 22 ( 50.0%) |  |
|  | | | |
| Survival Group |  |  | 0.94 |
| Short-term Survivors | 7 ( 46.7%) | 20 ( 45.5%) |  |
| Long-term Survivors | 8 ( 53.3%) | 24 ( 54.5%) |  |
|  | | | |
| *stratification factor | | | |
