# Supplementary material for: Genetic landscape of extreme responders with anaplastic oligodendroglioma
Source: Oncotarget. 2017 Mar 31;8(22):35523–31. doi: 10.18632/oncotarget.16773 (PMC5482595; doi:10.18632/oncotarget.16773)
Supplement: Supplementary file 2 [file oncotarget-08-35523-s002.docx]

| **Suplementary Table 2.** Pretreatment characteristics of patients in this tissue repository study (TRP206) compared to the remaining patients that were included in RTOG 9402 | | | |
| --- | --- | --- | --- |
|  | In TRP 206 (n=15) | Not in TRP 206 (n=276) | Chi-square  p-value |
|  | | | |
| Age* (years) |  |  | 0.18 |
| <50 | 8 ( 53.3%) | 193 ( 69.9%) |  |
| 50+ | 7 ( 46.7%) | 83 ( 30.1%) |  |
|  | | | |
| Gender |  |  | 0.98 |
| Male | 9 ( 60.0%) | 165 ( 59.8%) |  |
| Female | 6 ( 40.0%) | 111 ( 40.2%) |  |
|  | | | |
| Race |  |  | 0.07 |
| White | 12 ( 80.0%) | 249 ( 90.2%) |  |
| Hispanic | 0 ( 0.0%) | 9 ( 3.3%) |  |
| Black | 0 ( 0.0%) | 6 ( 2.2%) |  |
| Oriental | 2 ( 13.3%) | 5 ( 1.8%) |  |
| Native American | 0 ( 0.0%) | 1 ( 0.4%) |  |
| Other | 1 ( 6.7%) | 6 ( 2.2%) |  |
|  | | | |
| Karnofsky performance Status* |  |  | 0.63 |
| 60-70 | 1 ( 6.7%) | 29 ( 10.5%) |  |
| 80-100 | 14 ( 93.3%) | 247 ( 89.5%) |  |
|  | | | |
| Prior surgery |  |  | 0.89 |
| Biopsy | 1 ( 6.7%) | 34 ( 12.3%) |  |
| Partial Resection | 9 ( 60.0%) | 151 ( 54.7%) |  |
| Total Resection | 5 ( 33.3%) | 88 ( 31.9%) |  |
| Surgery done, no details | 0 ( 0.0%) | 3 ( 1.1%) |  |
|  | | | |
| Neurological function |  |  |  |
| No symptoms | 5 ( 33.3%) | 89 ( 32.2%) | 0.98 |
| Minor symptoms | 7 ( 46.7%) | 135 ( 48.9%) |  |
| Moderate (fully active) | 2 ( 13.3%) | 27 ( 9.8%) |  |
| Moderate (not fully active) | 1 ( 6.7%) | 24 ( 8.7%) |  |
| Unknown | 0 ( 0.0%) | 1 ( 0.4%) |  |
|  | | | |
| Histology |  |  | 0.08 |
| Anaplastic oligodendroglioma | 12 ( 80.0%) | 138 ( 50.0%) |  |
| Anaplastic oligoastrocytoma, oligo dominant | 3 ( 20.0%) | 62 ( 22.5%) |  |
| Anaplastic oligoastrocytoma, oligo=astro | 0 ( 0.0%) | 39 ( 14.1%) |  |
| Anaplastic oligoastrocytoma, astro dominant | 0 ( 0.0%) | 37 ( 13.4%) |  |
|  | | | |
| Grade* |  |  | 0.36 |
| Moderatly Anaplastic | 10 ( 66.7%) | 151 ( 54.7%) |  |
| Very Anaplastic | 5 ( 33.3%) | 125 ( 45.3%) |  |
|  | | | |
| *stratification factor | | | |
