# Supplementary material for: Genetic landscape of extreme responders with anaplastic oligodendroglioma
Source: Oncotarget. 2017 Mar 31;8(22):35523–31. doi: 10.18632/oncotarget.16773 (PMC5482595; doi:10.18632/oncotarget.16773)
Supplement: Supplementary file 3 [file oncotarget-08-35523-s003.docx]

| Sample ID | Gene Symbol | Gene Description | Transcript Accession | Nucleotide (genomic) | Amino Acid (protein) | Mutation Type | Consequence | Sequence Context (Position of Mutation Indicated by "N") | % Mutant Tags |
| --- | --- | --- | --- | --- | --- | --- | --- | --- | --- |
| LT-1 | ANK3 | ankyrin 3; node of Ranvier (ankyrin G) | CCDS7258.1 | chr10_61629895-61629895_T_G | 497K>Q | Substitution | Nonsynonymous coding | TACCTNAGCTT | 40% |
| LT-1 | ATRX | alpha thalassemia/mental retardation syndrome X-linked (RAD54 homolog; S. cerevisiae) | CCDS14434.1 | chrX_76826574-76826574_A_G | 277V>A | Substitution | Nonsynonymous coding | CAGTGNCCAAG | 45% |
| LT-1 | C3 | complement component 3 | CCDS32883.1 | chr19_6664419-6664419_G_A | 292P>L | Substitution | Nonsynonymous coding | GTACCNGAATG | 10% |
| LT-1 | C6orf70 | chromosome 6 open reading frame 70 | CCDS34576.1 | chr6_169917960-169917960_G_A | NA | Substitution | Splice site acceptor | TTTCANATTAG | 13% |
| LT-1 | CD38 | CD38 molecule | CCDS3417.1 | chr4_15435681-15435681_C_T | 148T>M | Substitution | Nonsynonymous coding | GGACANGCTGC | 36% |
| LT-1 | CIC | capicua homolog (Drosophila) | CCDS12601.1 | chr19_47485302-47485302_C_T | 422R>X | Substitution | Nonsense | CCACCNGAGCT | 80% |
| LT-1 | ESX1 | ESX homeobox 1 | CCDS14516.1 | chrX_103381827-103381827_G_C | 320P>R | Substitution | Nonsynonymous coding | GCACANGCGCC | 14% |
| LT-1 | EVL | Enah/Vasp-like | CCDS9955.1 | chr14_99673870-99673870_C_T | 358L>F | Substitution | Nonsynonymous coding | GCCCCNTTCAG | 13% |
| LT-1 | EVX2 | even-skipped homeobox 2 | CCDS33333.1 | chr2_176653496-176653496_C_T | 339R>H | Substitution | Nonsynonymous coding | GGTGGNGGAAG | 11% |
| LT-1 | IDH1 | isocitrate dehydrogenase 1 (NADP+); soluble | CCDS2381.1 | chr2_208821357-208821357_C_T | 132R>H | Substitution | Nonsynonymous coding | CATGANGACCT | 50% |
| LT-1 | KIAA0090 | uncharacterized protein | CCDS190.1 | chr1_19430377-19430377_C_T | 637A>T | Substitution | Nonsynonymous coding | CTTGGNGTAGT | 15% |
| LT-1 | LAMP2 | lysosomal-associated membrane protein 2 | CCDS14599.1 | chrX_119449307-119449307_G_C | 378P>A | Substitution | Nonsynonymous coding | TATGGNCACAA | 12% |
| LT-1 | MUC1 | mucin 1; cell surface associated | CCDS1098.1 | chr1_153426858-153426858_C_G | 147A>P | Substitution | Nonsynonymous coding | AGAGGNTGCTT | 29% |
| LT-1 | PCDH19 | protocadherin 19 | CCDS43976.1 | chrX_99483663-99483663_T_A | 867Q>H | Substitution | Nonsynonymous coding | TCAGTNTGGTC | 32% |
| LT-1 | PCDH8 | protocadherin 8 | CCDS9438.1 | chr13_52318562-52318562_T_C | 671I>V | Substitution | Nonsynonymous coding | CAGTANCTCCC | 45% |
| LT-1 | PIGC | phosphatidylinositol glycan anchor biosynthesis; class C | CCDS1302.1 | chr1_170677605-170677605_T_C | 261I>V | Substitution | Nonsynonymous coding | TGAGANAGACA | 38% |
| LT-1 | PJA1 | praja ring finger 1 | CCDS14393.1 | chrX_68299112-68299112_C_T | 232R>H | Substitution | Nonsynonymous coding | TGGGANGTGCA | 36% |
| LT-1 | PTCHD1 | patched domain containing 1 | CCDS35215.2 | chrX_23263113-23263113_G_A | 67R>H | Substitution | Nonsynonymous coding | CGAGCNCAACC | 18% |
| LT-1 | RIF1 | RAP1 interacting factor homolog (yeast) | CCDS2194.1 | chr2_152030491-152030491_G_C | 2071V>L | Substitution | Nonsynonymous coding | ACACANTTGAA | 16% |
| LT-1 | SLITRK6 | SLIT and NTRK-like family; member 6 | CCDS41903.1 | chr13_85266317-85266317_C_G | 776R>S | Substitution | Nonsynonymous coding | TTTTTNCTTAG | 15% |
| LT-2 | ABLIM2 | actin-binding LIM protein 2 isoform 1 | NM_001130083 | chr4_8113633-8113633_C_T | 242G>S | Substitution | Nonsynonymous coding | CTGGCNGCACC | 32% |
| LT-2 | AMICA1 | adhesion molecule; interacts with CXADR antigen 1 | CCDS41723.1 | chr11_117579574-117579574_C_T | 184R>H | Substitution | Nonsynonymous coding | AGTAANGAAAT | 44% |
| LT-2 | ANKK1 | ankyrin repeat and protein kinase domain-containing protein 1 | NM_178510 | chr11_112776059-112776059_G_A | 720V>I | Substitution | Nonsynonymous coding | TGGACNTCCAG | 43% |
| LT-2 | APC | adenomatous polyposis coli | CCDS4107.1 | chr5_112207697-112207697_G_C | 2836G>A | Substitution | Nonsynonymous coding | TTCTGNGTCTT | 47% |
| LT-2 | AUTS2 | autism susceptibility candidate 2 | CCDS5539.1 | chr7_69893893-69893893_C_T | 1252T>M | Substitution | Nonsynonymous coding | CCACANGCTGA | 35% |
| LT-2 | CCDC25 | coiled-coil domain containing 25 | CCDS6062.2 | chr8_27670165-27670165_C_T | 53R>Q | Substitution | Nonsynonymous coding | GTAATNGAAGG | 50% |
| LT-2 | CIC | capicua homolog (Drosophila) | CCDS12601.1 | chr19_47490900-47490900_G_A | 1515R>H | Substitution | Nonsynonymous coding | GGTGCNCCAGA | 65% |
| LT-2 | FAM83A | family with sequence similarity 83; member A | CCDS6340.1 | chr8_124288654-124288654_G_A | 284E>K | Substitution | Nonsynonymous coding | TTGACNAGGAG | 33% |
| LT-2 | FBN3 | fibrillin 3 | CCDS12196.1 | chr19_8097364-8097364_G_A | 848R>C | Substitution | Nonsynonymous coding | GCAGCNTTCGC | 32% |
| LT-2 | FLNB | filamin B; beta | CCDS2885.1 | chr3_58130468-58130468_C_T | 2510S>L | Substitution | Nonsynonymous coding | ATCCTNGGACG | 35% |
| LT-2 | FUBP1 | far upstream element (FUSE) binding protein 1 | CCDS683.1 | chr1_78193551-78193551_C_T | 586W>X | Substitution | Nonsense | CTTCCNAAGCC | 67% |
| LT-2 | IDH1 | isocitrate dehydrogenase 1 (NADP+); soluble | CCDS2381.1 | chr2_208821357-208821357_C_T | 132R>H | Substitution | Nonsynonymous coding | CATGACGACCT | 50% |
| LT-2 | KCNB2 | potassium voltage-gated channel; Shab-related subfamily; member 2 | CCDS6209.1 | chr8_73643019-73643019_G_A | 166E>K | Substitution | Nonsynonymous coding | GAGAANAGTTT | 38% |
| LT-2 | NEO1 | neogenin homolog 1 (chicken) | CCDS10247.1 | chr15_71380732-71380732__C | NA | Insertion | Frameshift | ACATTNACTCA | 30% |
| LT-2 | OR51B6 | olfactory receptor; family 51; subfamily B; member 6 | CCDS31379.1 | chr11_5329374-5329374_G_T | 21A>S | Substitution | Nonsynonymous coding | AGAAGNCACAT | 20% |
| LT-2 | OR51G2 | olfactory receptor; family 51; subfamily G; member 2 | CCDS31365.1 | chr11_4893346-4893346_C_G | 42V>L | Substitution | Nonsynonymous coding | GGAAANCAGAT | 29% |
| LT-2 | OR52E6 | olfactory receptor 52E6 | NM_001005167 | chr11_5819474-5819474_G_A | 77T>M | Substitution | Nonsynonymous coding | TGGCCNTAGAC | 27% |
| LT-2 | PANK3 | pantothenate kinase 3 | CCDS4368.1 | chr5_167923609-167923609_C_A | 225L>F | Substitution | Nonsynonymous coding | CCAGTNAATAA | 17% |
| LT-2 | PAQR4 | progestin and adipoQ receptor family member IV | CCDS10485.1 | chr16_2961568-2961568_C_T | 147P>L | Substitution | Nonsynonymous coding | GCGCCNGGCTG | 41% |
| LT-2 | PLEKHG2 | pleckstrin homology domain containing; family G (with RhoGef domain) member 2 | CCDS33022.1 | chr19_44597532-44597532_C_T | 15T>I | Substitution | Nonsynonymous coding | GAGCANAGTGG | 62% |
| LT-2 | PRPF4 | PRP4 pre-mRNA processing factor 4 homolog (yeast) | CCDS6791.1 | chr9_115092844-115092844_C_T | 398R>C | Substitution | Nonsynonymous coding | CAGGANGTTGT | 36% |
| LT-2 | PSMA3 | proteasome (prosome; macropain) subunit; alpha type; 3 | CCDS9731.1 | chr14_57784270-57784270_T_G | 24V>G | Substitution | Nonsynonymous coding | TCAAGNTGAAT | 31% |
| LT-2 | PTH2R | parathyroid hormone 2 receptor | CCDS2383.1 | chr2_208980072-208980072_T_C | 20L>P | Substitution | Nonsynonymous coding | CTGCCNCCTGG | 33% |
| LT-2 | RUSC1 | RUN and SH3 domain containing 1 | CCDS41410.1 | chr1_153561283-153561283_G_A | 461G>S | Substitution | Nonsynonymous coding | TCGCCNGTGTC | 41% |
| LT-2 | RXFP3 | relaxin/insulin-like family peptide receptor 3 | CCDS3900.1 | chr5_33973172-33973172_C_A | 190S>R | Substitution | Nonsynonymous coding | AAGAGNCACCG | 34% |
| LT-2 | SHROOM2 | shroom family member 2 | CCDS14135.1 | chrX_9823788-9823788_G_A | 614A>T | Substitution | Nonsynonymous coding | TCGACNCCCAC | 11% |
| LT-2 | SLC4A4 | solute carrier family 4; sodium bicarbonate cotransporter; member 4 | CCDS43236.1 | chr4_72551160-72551160_T_C | _ISV+2> | Substitution | Splice site donor | GCAAGNATGTA | 35% |
| LT-2 | SNX10 | sorting nexin 10 | CCDS5399.1 | chr7_26378054-26378054_G_A | 134V>I | Substitution | Nonsynonymous coding | CGTGTNTTTCT | 32% |
| LT-2 | SYNE1 | spectrin repeat containing; nuclear envelope 1 | CCDS5236.1 | chr6_152825675-152825675_G_T | 714T>K | Substitution | Nonsynonymous coding | AGTCTNTGTAT | 50% |
| LT-2 | TCHH | trichohyalin | CCDS41396.1 | chr1_150347477-150347477_G_A | 1614R>C | Substitution | Nonsynonymous coding | CTGGCNCAGCT | 31% |
| LT-2 | TESK2 | testis-specific kinase 2 | CCDS41323.1 | chr1_45583211-45583211_G_A | 535P>L | Substitution | Nonsynonymous coding | CCGCANGGCAT | 77% |
| LT-2 | TPD52 | tumor protein D52 | CCDS34912.1 | chr8_81126327-81126327_C_T | 166E>K | Substitution | Nonsynonymous coding | ATCTTNCAGCT | 50% |
| LT-3 | ABCC10 | ATP-binding cassette; sub-family C (CFTR/MRP); member 10 | CCDS4896.1 | chr6_43511952-43511952_G_A | 594E>K | Substitution | Nonsynonymous coding | ATCTCNAAGTG | 28% |
| LT-3 | ABCC12 | ATP-binding cassette; sub-family C (CFTR/MRP); member 12 | CCDS10730.1 | chr16_46719954-46719954_G_A | 478P>S | Substitution | Nonsynonymous coding | GGCTGNTGGAC | 15% |
| LT-3 | ATP2B1 | ATPase; Ca++ transporting; plasma membrane 1 | CCDS9035.1 | chr12_88517075-88517075_G_A | 1101R>W | Substitution | Nonsynonymous coding | ACGCCNCAACT | 37% |
| LT-3 | ATXN7L1 | ataxin 7-like 1 | ENST00000388807 | chr7_105045701-105045701_T_C | 91N>D | Substitution | Nonsynonymous coding | AATGTNGCTCA | 24% |
| LT-3 | CHRM5 | cholinergic receptor; muscarinic 5 | CCDS10031.1 | chr15_32142244-32142244_G_A | 12V>I | Substitution | Nonsynonymous coding | CCACCNTCAAT | 26% |
| LT-3 | EMR4 | Putative EGF-like module-containing mucin-like hormone receptor-like 4 precursor (G-protein coupled receptor 127) (G-protein coupled receptor PGR16). | ENST00000359590 | chr19_6922205-6922205_G_A | 258T>M | Substitution | Nonsynonymous coding | TGCCCNTCAGG | 31% |
| LT-3 | FAM48A | family with sequence similarity 48; member A | CCDS31959.1 | chr13_36493611-36493611_C_G | 597S>T | Substitution | Nonsynonymous coding | TTGCANTGGGC | 41% |
| LT-3 | FUBP1 | far upstream element (FUSE) binding protein 1 | CCDS683.1 | chr1_78205227-78205230_ACTA_ | NA | Deletion | Frameshift | CAATTNCTAGT | 64% |
| LT-3 | GCLC | glutamate-cysteine ligase; catalytic subunit | CCDS4952.1 | chr6_53480339-53480339_G_A | 328R>X | Substitution | Nonsense | ATATCNGGATT | 51% |
| LT-3 | HNRNPM | heterogeneous nuclear ribonucleoprotein M | CCDS12203.1 | chr19_8456640-8456640_G_A | 443R>H | Substitution | Nonsynonymous coding | GGACCNCATGG | 10% |
| LT-3 | HSP90AA1 | heat shock protein 90kDa alpha (cytosolic); class A member 1 | CCDS32160.1 | chr14_101619756-101619756_G_C | 578R>G | Substitution | Nonsynonymous coding | CTTCCNATTTT | 11% |
| LT-3 | HTRA1 | HtrA serine peptidase 1 | CCDS7630.1 | chr10_124259599-124259599_C_T | NA | Substitution | Splice site acceptor | CTCACNAGGAA | 10% |
| LT-3 | IDH1 | isocitrate dehydrogenase 1 (NADP+); soluble | CCDS2381.1 | chr2_208821357-208821357_C_T | 132R>H | Substitution | Nonsynonymous coding | CATGANGACCT | 54% |
| LT-3 | INTS5 | integrator complex subunit 5 | CCDS8027.1 | chr11_62173739-62173739_G_A | 130S>F | Substitution | Nonsynonymous coding | ACTCANACAGC | 10% |
| LT-3 | LGI4 | leucine-rich repeat LGI family; member 4 | CCDS12444.1 | chr19_40314208-40314208_C_T | 184A>T | Substitution | Nonsynonymous coding | ACAGGNGCCGG | 81% |
| LT-3 | LPIN1 | lipin 1 | CCDS1682.1 | chr2_11839952-11839952_T_G | 342F>V | Substitution | Nonsynonymous coding | TGCAGNTTGTG | 44% |
| LT-3 | MUSK | muscle, skeletal receptor tyrosine-protein kinase isoform 1 | NM_005592 | chr9_112602635-112602635_G_C | 719R>P | Substitution | Nonsynonymous coding | AGAACNTAAGT | 29% |
| LT-3 | MYH3 | myosin; heavy chain 3; skeletal muscle; embryonic | CCDS11157.1 | chr17_10482255-10482255_C_T | 1187E>K | Substitution | Nonsynonymous coding | GGCTTNGTGCT | 42% |
| LT-3 | NARS | asparaginyl-tRNA synthetase | CCDS32837.1 | chr18_53420665-53420665_T_C | 479I>V | Substitution | Nonsynonymous coding | AAAGANACGCA | 33% |
| LT-3 | NEB | nebulin isoform 1 | NM_001164507 | chr2_152221046-152221047_CT_ | NA | Deletion | Frameshift | TGACANTGAGC | 45% |
| LT-3 | NIPBL | Nipped-B homolog (Drosophila) | CCDS3920.1 | chr5_37021461-37021461_C_T | 808R>X | Substitution | Nonsense | ATGGGNGATCT | 41% |
| LT-3 | NOTCH1 | Notch homolog 1; translocation-associated (Drosophila) | CCDS43905.1 | chr9_138532891-138532893_AGA_ | NA | Deletion | In-frame deletion | GCAGTNGAAGG | 71% |
| LT-3 | NOTCH3 | Notch homolog 3 (Drosophila) | CCDS12326.1 | chr19_15133135-15133135_C_T | 2102V>M | Substitution | Nonsynonymous coding | GTCCANGGGCG | 15% |
| LT-3 | NRAP | nebulin-related anchoring protein | CCDS7579.1 | chr10_115370352-115370352_C_T | NA | Substitution | Splice site donor | TCTCANCTCGC | 10% |
| LT-3 | NRK | nik-related protein kinase | NM_198465 | chrX_105052702-105052702_A_T | 868D>V | Substitution | Nonsynonymous coding | GGTTGNCATCC | 30% |
| LT-3 | OR4K2 | olfactory receptor; family 4; subfamily K; member 2 | CCDS32023.1 | chr14_19414420-19414420_G_A | 52D>N | Substitution | Nonsynonymous coding | TAGTGNACCCT | 12% |
| LT-3 | PPP1R15A | protein phosphatase 1; regulatory (inhibitor) subunit 15A | CCDS12738.1 | chr19_54069903-54069903_G_A | 534R>Q | Substitution | Nonsynonymous coding | AAGGCNGCTCA | 18% |
| LT-3 | RBM11 | RNA binding motif protein 11 | NM_144770 | chr21_14521478-14521478_G_T | 280R>I | Substitution | Nonsynonymous coding | GAAAANATACT | 31% |
| LT-3 | SEPT6 | septin 6 | CCDS14584.1 | chrX_118655105-118655105_G_T | 290T>N | Substitution | Nonsynonymous coding | TGTGGNTCTGC | 28% |
| LT-3 | SFRS16 | splicing factor; arginine/serine-rich 16 | CCDS12652.2 | chr19_50255537-50255537_C_A | 254A>D | Substitution | Nonsynonymous coding | CAAGGNTCTTG | 15% |
| LT-3 | SIGLEC15 | sialic acid binding Ig-like lectin 15 | CCDS32819.1 | chr18_41672687-41672687__CGCGA | NA | Insertion | Frameshift | CGCGCNGCGGA | 20% |
| LT-3 | SPACA3 | sperm acrosome associated 3 | CCDS11275.1 | chr17_28348655-28348655_G_A | NA | Substitution | Splice site donor | TACTGNTAAGT | 12% |
| LT-3 | SPEF2 | sperm flagellar 2 | CCDS43309.1 | chr5_35736495-35736495_T_C | 761L>S | Substitution | Nonsynonymous coding | CACATNGGCTA | 46% |
| LT-3 | TNFRSF10C | tumor necrosis factor receptor superfamily; member 10c; decoy without an intracellular domain | CCDS6037.1 | chr8_23030395-23030395_T_C | 229I>T | Substitution | Nonsynonymous coding | AATGANCACCA | 42% |
| LT-3 | TNS4 | tensin 4 | CCDS11368.1 | chr17_35905887-35905887_T_C | 106N>S | Substitution | Nonsynonymous coding | TCTGANTGAGG | 53% |
| LT-3 | ZMYM4 | zinc finger; MYM-type 4 | CCDS389.1 | chr1_35608638-35608638_C_A | 335T>K | Substitution | Nonsynonymous coding | AGCCANAGCTG | 10% |
| LT-4 | ALPK3 | alpha-kinase 3 | CCDS10333.1 | chr15_83211562-83211562_G_A | 1780W>X | Substitution | Nonsense | GACTGNAAGAT | 32% |
| LT-4 | ALS2CR11 | amyotrophic lateral sclerosis 2 chromosomal region candidate gene 11 protein isoform 1 | NM_001168221 | chr2_202065885-202065885_C_G | 1142E>Q | Substitution | Nonsynonymous coding | TGACTNTGAGA | 37% |
| LT-4 | BICC1 | bicaudal C homolog 1 (Drosophila) | CCDS31206.1 | chr10_60247394-60247394_G_A | 867C>Y | Substitution | Nonsynonymous coding | TGGCTNTAACT | 24% |
| LT-4 | C1orf59 | chromosome 1 open reading frame 59 | CCDS787.1 | chr1_109001648-109001648_C_G | 33R>T | Substitution | Nonsynonymous coding | GCTGTNTGTAT | 11% |
| LT-4 | CDC42BPA | CDC42 binding protein kinase alpha (DMPK-like) | CCDS1558.1 | chr1_225288978-225288978_C_A | NA | Substitution | Splice site donor | TCTTANCTCAT | 38% |
| LT-4 | CDH19 | cadherin 19; type 2 | CCDS11994.1 | chr18_62348183-62348183_T_C | 446Y>C | Substitution | Nonsynonymous coding | TATTGNCTGCA | 38% |
| LT-4 | CIC | capicua homolog (Drosophila) | CCDS12601.1 | chr19_47483555-47483555_C_T | 201R>W | Substitution | Nonsynonymous coding | ACATCNGGCGG | 60% |
| LT-4 | CLK4 | CDC-like kinase 4 | CCDS4437.1 | chr5_177978296-177978296_T_C | 84Y>C | Substitution | Nonsynonymous coding | GAACANATCCT | 35% |
| LT-4 | CSNK2A1 | casein kinase 2; alpha 1 polypeptide | CCDS13003.1 | chr20_428554-428554_G_A | 80R>C | Substitution | Nonsynonymous coding | TTCACNCTTAA | 27% |
| LT-4 | CXorf36 | chromosome X open reading frame 36 | NM_176819 | chrX_44895866-44895866_T_G | 426D>A | Substitution | Nonsynonymous coding | TGCAANCTGGG | 34% |
| LT-4 | CXorf36 | chromosome X open reading frame 36 | NM_176819 | chrX_44895872-44895872_T_C | 424Y>C | Substitution | Nonsynonymous coding | CTGGGNAACGA | 32% |
| LT-4 | DNAH3 | dynein; axonemal; heavy chain 3 | CCDS10594.1 | chr16_20883576-20883576_G_A | 3044T>M | Substitution | Nonsynonymous coding | CTAACNTGTGG | 38% |
| LT-4 | FAM109A | family with sequence similarity 109; member A | ENST00000392658 | chr12_110284843-110284843__TT | NA | Insertion | Frameshift | CCCCCNAAAAC | 31% |
| LT-4 | FAM198A | hypothetical protein LOC729085 | NM_001129908 | chr3_43049582-43049582_G_A | 275G>R | Substitution | Nonsynonymous coding | CACAGNGGGAG | 33% |
| LT-4 | FAM65C | family with sequence similarity 65; member C | CCDS13431.2 | chr20_48669952-48669952_C_T | 79E>K | Substitution | Nonsynonymous coding | TGCTTNGAAGA | 16% |
| LT-4 | FGD5 | FYVE; RhoGEF and PH domain containing 5 | NM_152536 | chr3_14836363-14836363_G_A | 261G>R | Substitution | Nonsynonymous coding | TGGCCNGGGTC | 17% |
| LT-4 | FRAS1 | Fraser syndrome 1 | NM_025074 | chr4_79541054-79541054_A_G | 1373Y>C | Substitution | Nonsynonymous coding | AGACTNCCCCC | 41% |
| LT-4 | FUBP1 | far upstream element (FUSE) binding protein 1 | CCDS683.1 | chr1_78206446-78206446_G_A | 81Q>X | Substitution | Nonsense | ATTTTNAGGAG | 60% |
| LT-4 | GAA | glucosidase; alpha; acid | CCDS32760.1 | chr17_75707135-75707135_C_T | 912A>V | Substitution | Nonsynonymous coding | CACGGNGCCCC | 40% |
| LT-4 | HIRIP3 | HIRA interacting protein 3 | CCDS10664.1 | chr16_29913042-29913042_T_C | 309R>G | Substitution | Nonsynonymous coding | TTCTCNATCTC | 38% |
| LT-4 | HOXD3 | homeobox D3 | CCDS2270.1 | chr2_176744836-176744836_C_T | 296A>V | Substitution | Nonsynonymous coding | CGACGNGCCCT | 32% |
| LT-4 | HPCAL4 | hippocalcin like 4 | CCDS441.1 | chr1_39920881-39920881_C_T | 164D>N | Substitution | Nonsynonymous coding | CTGGTNGTCCT | 54% |
| LT-4 | HUWE1 | HECT; UBA and WWE domain containing 1 | CCDS35301.1 | chrX_53633330-53633330_G_A | 1455P>S | Substitution | Nonsynonymous coding | GTCTGNCAGCT | 28% |
| LT-4 | IDH1 | isocitrate dehydrogenase 1 (NADP+); soluble | CCDS2381.1 | chr2_208821357-208821357_C_T | 132R>H | Substitution | Nonsynonymous coding | CATGANGACCT | 35% |
| LT-4 | KCNA2 | potassium voltage-gated channel; shaker-related subfamily; member 2 | CCDS827.1 | chr1_110947670-110947670_C_ | NA | Deletion | Frameshift | TGTCTNCCGGT | 46% |
| LT-4 | KCNA2 | potassium voltage-gated channel; shaker-related subfamily; member 2 | CCDS827.1 | chr1_110947686-110947686_G_C | 414N>K | Substitution | Nonsynonymous coding | AAGTANTTGAA | 46% |
| LT-4 | KCNK17 | potassium channel; subfamily K; member 17 | CCDS4842.1 | chr6_39389932-39389932_G_A | 48A>V | Substitution | Nonsynonymous coding | CCTGCNCCGCG | 24% |
| LT-4 | LRFN2 | leucine rich repeat and fibronectin type III domain containing 2 | CCDS34443.1 | chr6_40507540-40507540_C_T | 431V>M | Substitution | Nonsynonymous coding | GGTCANTTCAG | 15% |
| LT-4 | MUC16 | mucin-16 | NM_024690 | chr19_8820691-8820691_G_A | 14481R>W | Substitution | Nonsynonymous coding | CTTCCNCCGGC | 17% |
| LT-4 | OTOG | otogelin | ENST00000399397 | chr11_17609963-17609963_G_A | 2223R>H | Substitution | Nonsynonymous coding | CTTCCNCCCAG | 28% |
| LT-4 | PACS1 | phosphofurin acidic cluster sorting protein 1 | CCDS8129.1 | chr11_65757855-65757855_C_T | 621P>L | Substitution | Nonsynonymous coding | CATGCNGAGGC | 31% |
| LT-4 | PCNX | pecanex homolog (Drosophila) | CCDS9806.1 | chr14_70514920-70514920_A_G | 705N>D | Substitution | Nonsynonymous coding | ACTCANACAGG | 52% |
| LT-4 | PIK3CA | phosphoinositide-3-kinase; catalytic; alpha polypeptide | CCDS43171.1 | chr3_180410674-180410674_T_C | 420C>R | Substitution | Nonsynonymous coding | AACACNGTCCA | 20% |
| LT-4 | PREX1 | phosphatidylinositol-3;4;5-trisphosphate-dependent Rac exchange factor 1 | CCDS13410.1 | chr20_46677864-46677864_C_T | 1604R>Q | Substitution | Nonsynonymous coding | GGCTCNGTGCC | 26% |
| LT-4 | PROKR2 | prokineticin receptor 2 | CCDS13089.1 | chr20_5230783-5230783_C_T | 353R>H | Substitution | Nonsynonymous coding | AGGGANGCCAG | 30% |
| LT-4 | SEMG2 | semenogelin II | CCDS13346.1 | chr20_43283965-43283965_C_T | 93A>V | Substitution | Nonsynonymous coding | TAAGGNGACAA | 42% |
| LT-4 | SLC25A24 | solute carrier family 25 (mitochondrial carrier; phosphate carrier); member 24 | CCDS41361.1 | chr1_108530065-108530065_C_T | 73G>E | Substitution | Nonsynonymous coding | GCTTCNCATCT | 28% |
| LT-4 | SLC39A6 | solute carrier family 39 (zinc transporter); member 6 | CCDS42428.1 | chr18_31948356-31948356_T_G | 515E>D | Substitution | Nonsynonymous coding | ACCTCNTCTTC | 30% |
| LT-4 | SPTBN5 | spectrin beta chain, brain 4 | NM_016642 | chr15_39931764-39931764_C_T | 3463G>R | Substitution | Nonsynonymous coding | TCTCCNGGGCT | 43% |
| LT-4 | SSTR2 | somatostatin receptor 2 | CCDS11691.1 | chr17_68677064-68677064_C_T | 4A>V | Substitution | Nonsynonymous coding | CATGGNGGATG | 34% |
| LT-4 | SYNPO2L | synaptopodin 2-like | CCDS7331.1 | chr10_75077315-75077315_C_T | 477V>M | Substitution | Nonsynonymous coding | TGTCANGTGAG | 33% |
| LT-4 | TRIP4 | thyroid hormone receptor interactor 4 | CCDS10194.1 | chr15_62480026-62480026_A_T | 217Q>L | Substitution | Nonsynonymous coding | TTTACNGCGTG | 23% |
| LT-4 | TRPM3 | transient receptor potential cation channel; subfamily M; member 3 | CCDS43835.1 | chr9_72340759-72340759_C_A | 1685G>V | Substitution | Nonsynonymous coding | TGTCCNCTCGG | 29% |
| LT-4 | TTC25 | tetratricopeptide repeat domain 25 | ENST00000377543 | chr17_37345517-37345517_A_G | 129N>S | Substitution | Nonsynonymous coding | CAACANCTCAG | 21% |
| LT-4 | ZKSCAN5 | zinc finger with KRAB and SCAN domains 5 | CCDS5667.1 | chr7_98967512-98967512_G_A | 742E>K | Substitution | Nonsynonymous coding | CAGCANAGAAG | 18% |
| LT-5 | ABCA13 | ATP-binding cassette; sub-family A (ABC1); member 13 | ENST00000319379 | chr7_48481719-48481719_T_C | 4263F>L | Substitution | Nonsynonymous coding | CAGAANTCCAG | 33% |
| LT-5 | AC005014.2 | Uncharacterized protein ENSP00000371558. | ENST00000401542 | chr7_16587604-16587604_G_T | 277E>X | Substitution | Nonsense | GCACANAAACA | 55% |
| LT-5 | CACNA2D2 | calcium channel; voltage-dependent; alpha 2/delta subunit 2 | CCDS33763.1 | chr3_50388311-50388311_C_T | 593R>H | Substitution | Nonsynonymous coding | TCCGANGGATC | 53% |
| LT-5 | CCDC18 | coiled-coil domain containing 18 | CCDS41356.1 | chr1_93470664-93470664_C_G | 914Q>E | Substitution | Nonsynonymous coding | AGCAANAGCAG | 22% |
| LT-5 | CD300A | CD300a molecule | CCDS32720.1 | chr17_69981435-69981435_G_A | 69G>E | Substitution | Nonsynonymous coding | AGCAGNAAAAA | 20% |
| LT-5 | CDHR3 | cadherin-related family member 3 precursor | NM_152750 | chr7_105449951-105449951_C_T | 633R>C | Substitution | Nonsynonymous coding | CATCTNGCTTT | 56% |
| LT-5 | CRYBG3 | beta-gamma crystallin domain containing 3 | CCDS43113.1 | chr3_99078874-99078874_T_C | 101V>A | Substitution | Nonsynonymous coding | CCTTGNCCATC | 44% |
| LT-5 | CYP27B1 | cytochrome P450; family 27; subfamily B; polypeptide 1 | CCDS8954.1 | chr12_56443710-56443710_C_G | 455C>S | Substitution | Nonsynonymous coding | CCATANAGCTG | 50% |
| LT-5 | DOK3 | docking protein 3 | CCDS4426.1 | chr5_176868110-176868110_C_T | 92G>S | Substitution | Nonsynonymous coding | TGGGCNTCCTG | 11% |
| LT-5 | EFCAB6 | EF-hand calcium binding domain 6 | CCDS14049.1 | chr22_42264662-42264662_G_A | 1326R>C | Substitution | Nonsynonymous coding | CTGGCNCCAGC | 36% |
| LT-5 | FAM171B | family with sequence similarity 171; member B | CCDS33347.1 | chr2_187326968-187326968_A_G | 320Y>C | Substitution | Nonsynonymous coding | GACATNTGATG | 41% |
| LT-5 | GLIPR1 | GLI pathogenesis-related 1 | CCDS9011.1 | chr12_74162043-74162043_G_A | 113A>T | Substitution | Nonsynonymous coding | CTTCCNCCATC | 35% |
| LT-5 | GPC3 | glypican 3 | CCDS14638.1 | chrX_132623455-132623458_TGAC_ | NA | Deletion | Frameshift | TAATTNGACTG | 16% |
| LT-5 | IDH1 | isocitrate dehydrogenase 1 (NADP+); soluble | CCDS2381.1 | chr2_208821357-208821357_C_T | 132R>H | Substitution | Nonsynonymous coding | CATGANGACCT | 43% |
| LT-5 | INPP1 | inositol polyphosphate-1-phosphatase | CCDS2305.1 | chr2_190944255-190944255_C_T | 361A>V | Substitution | Nonsynonymous coding | TGCTGNTGGGG | 13% |
| LT-5 | INSC | inscuteable homolog (Drosophila) | CCDS41621.1 | chr11_15217086-15217086_G_A | 475G>E | Substitution | Nonsynonymous coding | GTCTGNGACTC | 52% |
| LT-5 | KCNG3 | potassium voltage-gated channel; subfamily G; member 3 | CCDS1809.1 | chr2_42525101-42525101_G_A | 263T>M | Substitution | Nonsynonymous coding | ACGGCNTGATT | 31% |
| LT-5 | KIAA1324 | KIAA1324 | CCDS794.1 | chr1_109535905-109535905_C_T | 527T>M | Substitution | Nonsynonymous coding | GGAGANGTGGA | 14% |
| LT-5 | LCT | lactase | CCDS2178.1 | chr2_136283626-136283626_C_A | 921A>S | Substitution | Nonsynonymous coding | CCACGNGCCTT | 19% |
| LT-5 | LIPN | lipase; family member N | NM_001102469 | chr10_90512021-90512021_C_T | 69R>X | Substitution | Nonsense | ATGGGNGAACA | 44% |
| LT-5 | MAS1L | Mas-related G-protein coupled receptor MRG (MAS-R) (MAS1-like). | CCDS4661.1 | chr6_29562672-29562673_GA_ | NA | Deletion | Frameshift | ACTCTNAGAGA | 25% |
| LT-5 | MYB | v-myb myeloblastosis viral oncogene homolog (avian) | CCDS5174.1 | chr6_135548816-135548816_C_T | 36R>C | Substitution | Nonsynonymous coding | GAAAGNGTCAC | 19% |
| LT-5 | RAB11FIP5 | RAB11 family interacting protein 5 (class I) | CCDS1923.1 | chr2_73156285-73156285_G_A | 612R>W | Substitution | Nonsynonymous coding | CTCCCNCTGCA | 10% |
| LT-5 | RFX3 | regulatory factor X; 3 (influences HLA class II expression) | CCDS6449.1 | chr9_3283089-3283089_C_T | 240R>K | Substitution | Nonsynonymous coding | CCAATNTCCTG | 19% |
| LT-5 | SCFD2 | sec1 family domain containing 2 | CCDS33984.1 | chr4_53874644-53874644_T_G | 351K>Q | Substitution | Nonsynonymous coding | GTGCTNAGTGT | 11% |
| LT-5 | SCFD2 | sec1 family domain containing 2 | CCDS33984.1 | chr4_53874662-53874662_C_G | 345E>Q | Substitution | Nonsynonymous coding | AGCTTNCCATA | 12% |
| LT-5 | SF3B1 | splicing factor 3b; subunit 1; 155kDa | CCDS33356.1 | chr2_197981383-197981383_T_C | 358T>A | Substitution | Nonsynonymous coding | TGGTGNCTTTC | 54% |
| LT-5 | TJP3 | tight junction protein 3 (zona occludens 3) | CCDS32873.1 | chr19_3681525-3681525_G_A | 164R>H | Substitution | Nonsynonymous coding | CGAGCNCTCCC | 32% |
| LT-5 | TRIM59 | tripartite motif-containing 59 | CCDS3190.1 | chr3_161639034-161639034_C_G | 211G>A | Substitution | Nonsynonymous coding | GATTGNCAACA | 12% |
| LT-5 | USH2A | Usher syndrome 2A (autosomal recessive; mild) | CCDS31025.1 | chr1_214661973-214661973_G_C | 110T>R | Substitution | Nonsynonymous coding | CTGGTNTGATG | 14% |
| LT-5 | ZPBP | zona pellucida binding protein | CCDS5509.1 | chr7_49993607-49993607_G_A | 280R>C | Substitution | Nonsynonymous coding | TGCACNTCTGC | 16% |
| LT-6 | ABCA12 | ATP-binding cassette; sub-family A (ABC1); member 12 | CCDS33372.1 | chr2_215577205-215577205_C_A | 886E>X | Substitution | Nonsense | TAGTTNAACAG | 18% |
| LT-6 | ABCD2 | ATP-binding cassette; sub-family D (ALD); member 2 | CCDS8734.1 | chr12_38299645-38299645_A_G | 14W>R | Substitution | Nonsynonymous coding | GGTCCNTTTCA | 30% |
| LT-6 | AC005014.2 | Uncharacterized protein ENSP00000371558. | ENST00000401542 | chr7_16573565-16573565_C_A | 181S>X | Substitution | Nonsense | AAGATNAATGA | 16% |
| LT-6 | ACE | angiotensin I converting enzyme (peptidyl-dipeptidase A) 1 | CCDS11637.1 | chr17_58913701-58913701_G_A | 421G>R | Substitution | Nonsynonymous coding | CCATTNGGGAC | 48% |
| LT-6 | ACMSD | aminocarboxymuconate semialdehyde decarboxylase | CCDS2173.2 | chr2_135346560-135346560_G_A | 243R>H | Substitution | Nonsynonymous coding | CATGCNCCCAG | 15% |
| LT-6 | AGAP2 | ArfGAP with GTPase domain; ankyrin repeat and PH domain 2 | NM_001122772 | chr12_56418073-56418073_G_T | 75A>E | Substitution | Nonsynonymous coding | ACAGCNCATCC | 13% |
| LT-6 | ATP2A1 | ATPase; Ca++ transporting; cardiac muscle; fast twitch 1 | CCDS10643.1 | chr16_28798379-28798381_CTC_ | NA | Deletion | In-frame deletion | GGATTNTCCTC | 13% |
| LT-6 | ATP9B | ATPase; class II; type 9B | CCDS12014.1 | chr18_75138045-75138045_G_T | 424L>F | Substitution | Nonsynonymous coding | AGTTTNCGTGT | 42% |
| LT-6 | C10orf62 | chromosome 10 open reading frame 62 | CCDS31261.1 | chr10_99340138-99340140_AGG_ | NA | Deletion | In-frame deletion | CAACCNGGAGG | 15% |
| LT-6 | CAMK2N2 | calcium/calmodulin-dependent protein kinase II inhibitor 2 | CCDS3257.1 | chr3_185461649-185461649_T_C | 40N>S | Substitution | Nonsynonymous coding | CCTGGNTGCCC | 39% |
| LT-6 | CCDC17 | coiled-coil domain-containing protein 17 isoform 1 | NM_001114938 | chr1_45858592-45858596_TCCTT_ | NA | Deletion | Frameshift | CTCTANCCTTG | 20% |
| LT-6 | CHD2 | chromodomain helicase DNA binding protein 2 | CCDS10374.2 | chr15_91300781-91300784_TGAT_ | NA | Deletion | Frameshift | AACTCNGATTG | 31% |
| LT-6 | CIC | capicua homolog (Drosophila) | CCDS12601.1 | chr19_47483212-47483213_AG_ | NA | Deletion | Frameshift | GAGACNGAGAG | 26% |
| LT-6 | CIC | capicua homolog (Drosophila) | CCDS12601.1 | chr19_47486974-47486975_CT_ | NA | Deletion | Frameshift | GCCACNTCACC | 34% |
| LT-6 | CREBZF | CREB/ATF bZIP transcription factor | CCDS41697.1 | chr11_85052890-85052892_CTT_ | NA | Deletion | In-frame deletion | TACTCNTTCTT | 18% |
| LT-6 | CTTNBP2 | cortactin binding protein 2 | CCDS5774.1 | chr7_117138887-117138889_GTT_ | NA | Deletion | In-frame deletion | TTTGANTTGTT | 20% |
| LT-6 | DHX36 | DEAH (Asp-Glu-Ala-His) box polypeptide 36 | CCDS3171.1 | chr3_155485107-155485107_C_T | 751D>N | Substitution | Nonsynonymous coding | AGTATNCTTTG | 40% |
| LT-6 | DTNBP1 | dystrobrevin binding protein 1 | CCDS4534.1 | chr6_15735636-15735637_TT_ | NA | Deletion | Frameshift | TTGTCNTTTTC | 24% |
| LT-6 | EEF1G | eukaryotic translation elongation factor 1 gamma | CCDS8021.1 | chr11_62099411-62099414_GCAA_ | NA | Deletion | Frameshift | CTGCTNCAAGC | 12% |
| LT-6 | EIF3L | eukaryotic translation initiation factor 3; subunit L | CCDS13960.1 | chr22_36575424-36575425_TA_ | NA | Deletion | Frameshift | ATGATNATGAG | 35% |
| LT-6 | EPHA7 | EPH receptor A7 | CCDS5031.1 | chr6_94181206-94181206_A_T | 33V>E | Substitution | Nonsynonymous coding | GTAGTNCTGAA | 42% |
| LT-6 | ESX1 | ESX homeobox 1 | CCDS14516.1 | chrX_103381846-103381846_T_G | 314T>P | Substitution | Nonsynonymous coding | CCCGGNTGGCA | 30% |
| LT-6 | FAM83A | family with sequence similarity 83; member A | CCDS6340.1 | chr8_124288789-124288789_C_T | 329R>C | Substitution | Nonsynonymous coding | GTGACNGCACG | 10% |
| LT-6 | FOXP1 | forkhead box P1 | CCDS2914.1 | chr3_71119872-71119872_T_C | 370H>R | Substitution | Nonsynonymous coding | TCACANGCAGG | 51% |
| LT-6 | FRY | furry homolog (Drosophila) | CCDS41875.1 | chr13_31589539-31589539_G_A | 131W>X | Substitution | Nonsense | GACTGNTATAA | 19% |
| LT-6 | HDAC1 | histone deacetylase 1 | CCDS360.1 | chr1_32570418-32570420_GAG_ | NA | Deletion | In-frame deletion | ACCCANAGGAG | 24% |
| LT-6 | HEG1 | HEG homolog 1 (zebrafish) | NM_020733 | chr3_126172198-126172200_TCT_ | NA | Deletion | In-frame deletion | TAGTCNCTTCT | 34% |
| LT-6 | HPCAL1 | hippocalcin-like 1 | CCDS1671.1 | chr2_10477530-10477530_G_A | 66E>K | Substitution | Nonsynonymous coding | TCGCCNAGCAC | 14% |
| LT-6 | IGLV10-54 | immunoglobulin lambda variable 10-54 | ENST00000390287 | chr22_20899490-20899490_G_A | 61G>D | Substitution | Nonsynonymous coding | CCAGGNCCACC | 24% |
| LT-6 | INPP4B | inositol polyphosphate-4-phosphatase; type II; 105kDa | CCDS3757.1 | chr4_143286464-143286464_C_A | 567A>S | Substitution | Nonsynonymous coding | AATGGNATCTG | 23% |
| LT-6 | ITGAD | integrin; alpha D | CCDS32438.1 | chr16_31330018-31330018_G_A | 493V>M | Substitution | Nonsynonymous coding | TGTCCNTGTGT | 50% |
| LT-6 | KAZALD1 | Kazal-type serine peptidase inhibitor domain 1 | CCDS7509.1 | chr10_102812797-102812797_G_A | 153R>H | Substitution | Nonsynonymous coding | GGCCCNCGCTC | 37% |
| LT-6 | KIAA2026 | putative uncharacterized protein ENSP00000382813 | NM_001017969 | chr9_5944057-5944059_TCT_ | NA | Deletion | In-frame deletion | TTTCCNCTTCT | 29% |
| LT-6 | KIDINS220 | kinase D-interacting substrate; 220kDa | CCDS42650.1 | chr2_8843591-8843591_T_ | NA | Deletion | Frameshift | ACATGNTTTTT | 11% |
| LT-6 | MAB21L2 | mab-21-like 2 (C. elegans) | CCDS3774.1 | chr4_151723726-151723727_GA_ | NA | Deletion | Frameshift | CATCCNAGAGG | 34% |
| LT-6 | MCM8 | minichromosome maintenance complex component 8 | CCDS13094.1 | chr20_5896212-5896213_AA_ | NA | Deletion | Frameshift | TTGTCNAAGTC | 33% |
| LT-6 | MGMT | methylated-DNA--protein-cysteine methyltransferase | NM_002412 | chr10_131155479-131155479_C_A | 4Q>K | Substitution | Nonsynonymous coding | TGGGANAGCCC | 12% |
| LT-6 | MLL | myeloid/lymphoid or mixed-lineage leukemia (trithorax homolog; Drosophila) | CCDS31686.1 | chr11_117849582-117849584_CTC_ | NA | Deletion | In-frame deletion | TCCTANTCCTC | 32% |
| LT-6 | MLL2 | histone-lysine N-methyltransferase MLL2 | NM_003482 | chr12_47719508-47719508_G_C | 2736Q>E | Substitution | Nonsynonymous coding | GGTCTNGCCTC | 16% |
| LT-6 | MTSS1L | metastasis suppressor 1-like | CCDS32476.1 | chr16_69255623-69255624_GT_ | NA | Deletion | Frameshift | GGAGGNTGTGC | 30% |
| LT-6 | MUC17 | mucin 17; cell surface associated | CCDS34711.1 | chr7_100483127-100483127_G_A | 4415R>H | Substitution | Nonsynonymous coding | TTTCCNCTCCA | 39% |
| LT-6 | MYO15A | myosin XVA | CCDS42271.1 | chr17_18005357-18005357_G_A | 3130D>N | Substitution | Nonsynonymous coding | GCAGGNACAGC | 42% |
| LT-6 | NIPBL | Nipped-B homolog (Drosophila) | CCDS3920.1 | chr5_37081360-37081363_AACA_ | NA | Deletion | Frameshift | CTTCTNACAAA | 25% |
| LT-6 | NLRP11 | NLR family; pyrin domain containing 11 | CCDS12935.1 | chr19_60988858-60988858_G_A | 1016P>L | Substitution | Nonsynonymous coding | TTCTGNGAAAT | 12% |
| LT-6 | NOS1 | nitric oxide synthase 1 (neuronal) | CCDS41842.1 | chr12_116252896-116252896_C_T | 121R>Q | Substitution | Nonsynonymous coding | TCACCNGGATG | 47% |
| LT-6 | NOTCH1 | Notch homolog 1; translocation-associated (Drosophila) | CCDS43905.1 | chr9_138532891-138532893_AGA_ | NA | Deletion | In-frame deletion | GCAGTNGAAGG | 38% |
| LT-6 | NOTCH1 | Notch homolog 1; translocation-associated (Drosophila) | CCDS43905.1 | chr9_138538190-138538190_C_T | 68C>Y | Substitution | Nonsynonymous coding | TCTTGNAGGGG | 21% |
| LT-6 | PCNXL2 | pecanex-like 2 (Drosophila) | NM_014801 | chr1_231420432-231420432_C_T | 876V>I | Substitution | Nonsynonymous coding | CATGANGAGGC | 14% |
| LT-6 | PIK3AP1 | phosphoinositide-3-kinase adaptor protein 1 | CCDS31259.1 | chr10_98376592-98376594_TCT_ | NA | Deletion | In-frame deletion | AAACANCTTCT | 14% |
| LT-6 | PLSCR4 | phospholipid scramblase 4 | CCDS3133.1 | chr3_147400289-147400289_C_T | NA | Substitution | Splice site donor | TCTGANCTCTT | 42% |
| LT-6 | PRPF19 | PRP19/PSO4 pre-mRNA processing factor 19 homolog (S. cerevisiae) | CCDS7995.1 | chr11_60415314-60415316_GAG_ | NA | Deletion | Splice site acceptor | GCTCTNAGGAG | 15% |
| LT-6 | PTPN13 | tyrosine-protein phosphatase non-receptor type 13 isoform 4 | NM_080685 | chr4_87947927-87947929_AGA_ | NA | Deletion | In-frame deletion | GAAGTNGAAGG | 26% |
| LT-6 | PTPRZ1 | protein tyrosine phosphatase; receptor-type; Z polypeptide 1 | CCDS34740.1 | chr7_121440760-121440763_ACTC_ | NA | Deletion | Frameshift | CTCATNCTCAC | 22% |
| LT-6 | RAPH1 | Ras association (RalGDS/AF-6) and pleckstrin homology domains 1 | CCDS2360.1 | chr2_204043247-204043249_AGA_ | NA | Deletion | In-frame deletion | TTCTCNGAAGA | 32% |
| LT-6 | RBPMS2 | RNA binding protein with multiple splicing 2 | CCDS32271.1 | chr15_62827768-62827768_G_A | 157P>L | Substitution | Nonsynonymous coding | GGTAGNGGGCC | 19% |
| LT-6 | RELL2 | RELT-like 2 | CCDS4265.1 | chr5_140998094-140998096_AAG_ | NA | Deletion | In-frame deletion | TGCTCNAGAAG | 14% |
| LT-6 | RLBP1L2 | Retinaldehyde-binding protein 1-like protein 2. | CCDS34525.1 | chr6_123360716-123360718_AGG_ | NA | Deletion | In-frame deletion | CATCCNGGAGG | 38% |
| LT-6 | RRH | retinal pigment epithelium-derived rhodopsin homolog | CCDS3687.1 | chr4_110983156-110983156_C_A | 268P>Q | Substitution | Nonsynonymous coding | TGACCNAAAGA | 10% |
| LT-6 | SCN10A | sodium channel; voltage-gated; type X; alpha subunit | CCDS33736.1 | chr3_38768979-38768979_C_T | 497R>H | Substitution | Nonsynonymous coding | CCCGGNGTTTT | 47% |
| LT-6 | SETD2 | SET domain containing 2 | CCDS2749.1 | chr3_47100793-47100793_C_A | 1324W>C | Substitution | Nonsynonymous coding | TGAGANCAGCG | 25% |
| LT-6 | SETD2 | SET domain containing 2 | NM_014159 | chr3_47180341-47180344_TACT_ | NA | Deletion | Splice site donor | GCTGANACTTA | 32% |
| LT-6 | SETMAR | SET domain and mariner transposase fusion gene | CCDS2563.1 | chr3_4330187-4330189_AGA_ | NA | Deletion | In-frame deletion | GTGCCNGAAGA | 32% |
| LT-6 | SLC38A3 | sodium-coupled neutral amino acid transporter 3 | NM_006841 | chr3_50232609-50232609_A_G | 504H>R | Substitution | Nonsynonymous coding | AAACCNCTAGG | 40% |
| LT-6 | SMOC1 | SPARC related modular calcium binding 1 | CCDS32110.1 | chr14_69489960-69489961_TG_ | NA | Deletion | Frameshift | GAAGCNGTGTT | 30% |
| LT-6 | SORCS3 | sortilin-related VPS10 domain containing receptor 3 | CCDS7558.1 | chr10_106906977-106906977_G_A | 525R>H | Substitution | Nonsynonymous coding | TTGGCNCCTGC | 13% |
| LT-6 | SPG21 | spastic paraplegia 21 (autosomal recessive; Mast syndrome) | CCDS10198.1 | chr15_63060398-63060398_C_A | 28D>Y | Substitution | Nonsynonymous coding | ACTGTNATCAT | 10% |
| LT-6 | SSPO | SCO-spondin precursor | NM_198455 | chr7_149133561-149133561_G_C | 2815R>S | Substitution | Nonsynonymous coding | CCCAGNAGATG | 12% |
| LT-6 | STAT4 | signal transducer and activator of transcription 4 | ENST00000409995 | chr2_191704834-191704834_C_G | 104R>P | Substitution | Nonsynonymous coding | CATGTNGATCC | 50% |
| LT-6 | SUN2 | Sad1 and UNC84 domain containing 2 | CCDS13978.1 | chr22_37464532-37464535_ACTC_ | NA | Deletion | Splice site donor | CAGATNCTCAC | 16% |
| LT-6 | TFDP3 | transcription factor Dp family; member 3 | NM_016521 | chrX_132179712-132179712_G_A | 81T>I | Substitution | Nonsynonymous coding | AGTGANTGCTG | 44% |
| LT-6 | TGM5 | transglutaminase 5 | CCDS32212.1 | chr15_41340073-41340073_C_T | NA | Substitution | Splice site acceptor | CCCTGNAATGG | 26% |
| LT-6 | TIAM1 | T-cell lymphoma invasion and metastasis 1 | CCDS13609.1 | chr21_31481238-31481238_C_T | 871R>Q | Substitution | Nonsynonymous coding | GCCTTNGAATA | 80% |
| LT-6 | TP53 | tumor protein p53 | CCDS11118.1 | chr17_7518938-7518939_AA_ | NA | Deletion | Frameshift | TGTCGNAAAGT | 9% |
| LT-6 | TRANK1 | TPR and ankyrin repeat-containing protein 1 | NM_014831 | chr3_36890658-36890658_G_T | 303H>Q | Substitution | Nonsynonymous coding | TCTAANTGACT | 14% |
| LT-6 | TYRP1 | tyrosinase-related protein 1 | CCDS34990.1 | chr9_12692411-12692414_ACAA_ | NA | Deletion | Frameshift | ACTCTNCAAAC | 35% |
| LT-6 | UBR5 | ubiquitin protein ligase E3 component n-recognin 5 | CCDS34933.1 | chr8_103350448-103350450_AGA_ | NA | Deletion | In-frame deletion | TTGCTNGAAGG | 26% |
| LT-6 | USP43 | ubiquitin carboxyl-terminal hydrolase 43 | NM_153210 | chr17_9572832-9572832_C_T | 1058R>W | Substitution | Nonsynonymous coding | GCAGCNGGCTC | 47% |
| LT-6 | VASN | vasorin | CCDS10514.1 | chr16_4371975-4371975_C_T | 366R>W | Substitution | Nonsynonymous coding | TGGTGNGGGAG | 37% |
| LT-6 | VAV1 | vav 1 guanine nucleotide exchange factor | CCDS12174.1 | chr19_6779455-6779455_C_T | 350A>V | Substitution | Nonsynonymous coding | GGAGGNGATGG | 26% |
| LT-6 | ZBTB16 | zinc finger and BTB domain containing 16 | CCDS8367.1 | chr11_113440086-113440086_G_A | 285R>Q | Substitution | Nonsynonymous coding | GACTCNAAGCA | 42% |
| LT-6 | ZDHHC20 | probable palmitoyltransferase ZDHHC20 | NM_153251 | chr13_20885830-20885830_C_T | 111A>T | Substitution | Nonsynonymous coding | TCTTGNTGCTC | 33% |
| LT-6 | ZFPM1 | zinc finger protein; multitype 1 | CCDS32502.1 | chr16_87126725-87126727_CCA_ | NA | Deletion | In-frame deletion | CATCTNCACCA | 29% |
| LT-6 | ZNF18 | zinc finger protein 18 | CCDS32568.1 | chr17_11828160-11828160_G_T | 249S>X | Substitution | Nonsense | CTCCTNAGACC | 15% |
| LT-6 | ZNF37A | zinc finger protein 37A | CCDS31183.1 | chr10_38444129-38444129_G_A | 48G>E | Substitution | Nonsynonymous coding | CACAGNGTATT | 43% |
| LT-7 | AP002478.3 | cDNA FLJ35776 fis, clone TESTI2005326. | ENST00000317114 | chr18_3587036-3587036_C_ | NA | Deletion | Frameshift | CACTGNCCTTT | 48% |
| LT-7 | AURKA | aurora kinase A | CCDS13451.1 | chr20_54394872-54394872_C_T | 56R>H | Substitution | Nonsynonymous coding | GAATGNGCTGG | 28% |
| LT-7 | C1orf204 | chromosome 1 open reading frame 204 | CCDS30913.1 | chr1_158098920-158098922_CTC_ | NA | Deletion | In-frame deletion | GAATGNTCCTC | 28% |
| LT-7 | C1orf56 | chromosome 1 open reading frame 56 | CCDS980.1 | chr1_149287952-149287955_AGTA_ | NA | Deletion | Splice site donor | CAACCNGTAAG | 32% |
| LT-7 | CDKN2C | cyclin-dependent kinase inhibitor 2C (p18; inhibits CDK4) | CCDS555.1 | chr1_51208633-51208633__C | NA | Insertion | Frameshift | AATGGNCGAGC | 55% |
| LT-7 | CIC | capicua homolog (Drosophila) | CCDS12601.1 | chr19_47487557-47487557__A | NA | Insertion | Frameshift | CAGTCNGTTTC | 62% |
| LT-7 | CSMD1 | CUB and sushi domain-containing protein 1 precursor | NM_033225 | chr8_2810713-2810713_C_A | 3091C>F | Substitution | Nonsynonymous coding | GCAGANAGGTT | 50% |
| LT-7 | EPYC | epiphycan | CCDS31870.1 | chr12_89882164-89882164_G_T | 290A>E | Substitution | Nonsynonymous coding | CTAGTNCCTTA | 34% |
| LT-7 | FAM181B | family with sequence similarity 181; member B | CCDS31648.1 | chr11_82121545-82121545_G_A | 292A>V | Substitution | Nonsynonymous coding | CTCCGNCGGGG | 13% |
| LT-7 | GPR111 | G protein-coupled receptor 111 | CCDS43471.1 | chr6_47757815-47757815_G_A | 453A>T | Substitution | Nonsynonymous coding | TGGCANCCACA | 44% |
| LT-7 | HDAC2 | histone deacetylase 2 | CCDS43493.1 | chr6_114381234-114381234_T_C | 274H>R | Substitution | Nonsynonymous coding | CACCANGATGA | 28% |
| LT-7 | IDH1 | isocitrate dehydrogenase 1 (NADP+); soluble | CCDS2381.1 | chr2_208821357-208821357_C_T | 132R>H | Substitution | Nonsynonymous coding | CATGANGACCT | 21% |
| LT-7 | IQSEC3 | IQ motif and Sec7 domain 3 | CCDS31725.1 | chr12_150731-150731_A_G | 755I>V | Substitution | Nonsynonymous coding | ATACANTTAAA | 44% |
| LT-7 | MAX | MYC associated factor X | CCDS9771.1 | chr14_64614500-64614500_C_T | 60R>Q | Substitution | Nonsynonymous coding | GGGCCNGGGAT | 44% |
| LT-7 | OR5D16 | olfactory receptor; family 5; subfamily D; member 16 | CCDS31512.1 | chr11_55363629-55363629_G_T | 276A>S | Substitution | Nonsynonymous coding | AAGTGNCCTCT | 18% |
| LT-7 | PNMA5 | paraneoplastic antigen like 5 | CCDS14718.1 | chrX_151910285-151910285_C_T | 172E>K | Substitution | Nonsynonymous coding | CTCTTNGCCTG | 15% |
| LT-7 | POLR3B | polymerase (RNA) III (DNA directed) polypeptide B | CCDS9105.1 | chr12_105375125-105375125_G_A | 791M>I | Substitution | Nonsynonymous coding | GTGATNGGGCC | 45% |
| LT-7 | PPP2R3A | protein phosphatase 2 (formerly 2A); regulatory subunit B''; alpha | CCDS3087.1 | chr3_137303631-137303631_G_A | 1007R>Q | Substitution | Nonsynonymous coding | TGAACNGATGG | 43% |
| LT-7 | RGS11 | regulator of G-protein signaling 11 | CCDS42088.1 | chr16_261233-261236_TGTC_ | NA | Deletion | Frameshift | GTCATNGTCTG | 26% |
| LT-7 | RNF214 | ring finger protein 214 | CCDS41720.1 | chr11_116657562-116657562_G_A | 425G>R | Substitution | Nonsynonymous coding | GGAACNGAGCC | 39% |
| LT-7 | SH2D3A | SH2 domain containing 3A | CCDS12173.1 | chr19_6711707-6711707_G_A | 121R>X | Substitution | Nonsense | GCGTCNCAGAG | 38% |
| LT-7 | SP110 | SP110 nuclear body protein | CCDS2475.1 | chr2_230787962-230787962_A_G | 88I>T | Substitution | Nonsynonymous coding | GGTTANTTTGA | 29% |
| LT-7 | SREBF1 | sterol regulatory element binding transcription factor 1 | CCDS32583.1 | chr17_17662295-17662295_T_A | NA | Substitution | Splice site donor | AGGACNCACTG | 35% |
| LT-7 | TTN | titin | ENST00000375038 | chr2_179137008-179137008_A_G | 23347V>A | Substitution | Nonsynonymous coding | CAAGTNCCTTT | 34% |
| LT-8 | ABCA10 | ATP-binding cassette; sub-family A (ABC1); member 10 | CCDS11684.1 | chr17_64663651-64663651_G_A | 1156R>W | Substitution | Nonsynonymous coding | ACTCCNTGGAG | 24% |
| LT-8 | AC026740.6 | Uncharacterized protein ENSP00000341823. | ENST00000343883 | chr5_849352-849352_A_G | 81C>R | Substitution | Nonsynonymous coding | TAAGCNGCAAG | 35% |
| LT-8 | CCDC40 | coiled-coil domain containing 40 | CCDS42395.1 | chr17_75676159-75676159_C_T | 870R>C | Substitution | Nonsynonymous coding | TCGTGNGCTCG | 17% |
| LT-8 | CIC | capicua homolog (Drosophila) | CCDS12601.1 | chr19_47488171-47488171_C_T | 994Q>X | Substitution | Nonsense | AGCCTNAGAAG | 44% |
| LT-8 | CRX | cone-rod homeobox | CCDS12706.1 | chr19_53034701-53034701_G_A | 189A>T | Substitution | Nonsynonymous coding | CCTCCNCCCCC | 50% |
| LT-8 | DBR1 | debranching enzyme homolog 1 (S. cerevisiae) | CCDS33863.1 | chr3_139373219-139373219_C_T | 117G>S | Substitution | Nonsynonymous coding | TACACNTCGGT | 35% |
| LT-8 | DHX16 | Putative pre-mRNA-splicing factor ATP-dependent RNA helicase DHX16 | CCDS4685.1 | chr6_30748581-30748581_C_T | 6G>D | Substitution | Nonsynonymous coding | CCAGANCCGCC | 27% |
| LT-8 | ECSCR | endothelial cell-specific chemotaxis regulator precursor | NM_001077693 | chr5_138817924-138817924_C_A | 30Q>H | Substitution | Nonsynonymous coding | GAGGTNTGGGT | 12% |
| LT-8 | ERVFRD-1 | HERV-FRD_6p24.1 provirus ancestral Env polyprotein preproprotein | NM_207582 | chr6_11212961-11212961_C_T | 190S>N | Substitution | Nonsynonymous coding | ACCGGNTGGAT | 11% |
| LT-8 | ESX1 | ESX homeobox 1 | CCDS14516.1 | chrX_103381846-103381846_T_G | 314T>P | Substitution | Nonsynonymous coding | CCCGGNTGGCA | 16% |
| LT-8 | FUBP1 | far upstream element (FUSE) binding protein 1 | CCDS683.1 | chr1_78203343-78203343_G_A | 212Q>X | Substitution | Nonsense | TACCTNAAGCT | 50% |
| LT-8 | FZD4 | frizzled homolog 4 (Drosophila) | CCDS8279.1 | chr11_86341163-86341163_G_A | NA | Substitution | Splice site acceptor | GAACTNGAAAA | 55% |
| LT-8 | IDH1 | isocitrate dehydrogenase 1 (NADP+); soluble | CCDS2381.1 | chr2_208821357-208821357_C_T | 132R>H | Substitution | Nonsynonymous coding | CATGANGACCT | 0.6 |
| LT-8 | INTS9 | integrator complex subunit 9 | CCDS34873.1 | chr8_28707350-28707350_C_A | 310E>D | Substitution | Nonsynonymous coding | AGGCANTCCAG | 11% |
| LT-8 | KIAA1109 | KIAA1109 | CCDS43267.1 | chr4_123488222-123488222_C_T | 4323P>S | Substitution | Nonsynonymous coding | AGCAGNCAAGT | 21% |
| LT-8 | LENG8 | leukocyte receptor cluster (LRC) member 8 | CCDS12894.1 | chr19_59658452-59658452_G_A | 307E>K | Substitution | Nonsynonymous coding | AGTCGNAGGAG | 43% |
| LT-8 | MFSD2A | major facilitator superfamily domain containing 2A | CCDS446.1 | chr1_40203752-40203752_C_T | 167A>V | Substitution | Nonsynonymous coding | CTCGGNTCTCA | 59% |
| LT-8 | OR10Q1 | olfactory receptor; family 10; subfamily Q; member 1 | CCDS31547.1 | chr11_57752499-57752499_C_T | 142R>H | Substitution | Nonsynonymous coding | GCTCGNGGGTC | 25% |
| LT-8 | PAOX | polyamine oxidase (exo-N4-amino) | CCDS7683.1 | chr10_135053233-135053233_G_A | 462G>S | Substitution | Nonsynonymous coding | GCGCCNGCGCC | 35% |
| LT-8 | PCDH12 | protocadherin 12 | CCDS4269.1 | chr5_141316351-141316351_G_A | 417T>I | Substitution | Nonsynonymous coding | CCAGTNTGGCA | 13% |
| LT-8 | PQBP1 | polyglutamine binding protein 1 | CCDS14309.1 | chrX_48645012-48645012_C_T | 213P>S | Substitution | Nonsynonymous coding | ACGCCNCCCGG | 18% |
| LT-8 | ProSAPiP1 | Uncharacterized protein KIAA0552. | CCDS13049.1 | chr20_3095634-3095634_G_A | 59T>I | Substitution | Nonsynonymous coding | CCCCTNTGCGG | 17% |
| LT-8 | PRPF4B | PRP4 pre-mRNA processing factor 4 homolog B (yeast) | CCDS4488.1 | chr6_3994994-3994994_C_T | 686R>C | Substitution | Nonsynonymous coding | ATAAANGTTAC | 29% |
| LT-8 | PUM2 | pumilio homolog 2 (Drosophila) | CCDS1698.1 | chr2_20390593-20390593_C_A | 4D>Y | Substitution | Nonsynonymous coding | AAAATNATGAT | 13% |
| LT-8 | QDPR | quinoid dihydropteridine reductase | CCDS3421.1 | chr4_17115127-17115127_A_T | 90W>R | Substitution | Nonsynonymous coding | GGCCCNTCCTC | 25% |
| LT-8 | REG3G | regenerating islet-derived 3 gamma | CCDS1962.1 | chr2_79107440-79107440_C_A | 57S>X | Substitution | Nonsense | TTTGTNACCAA | 11% |
| LT-8 | RPL24 | ribosomal protein L24 | CCDS33809.1 | chr3_102884006-102884006_T_ | NA | Deletion | Frameshift | TAGCCNTTTTT | 10% |
| LT-8 | SLC15A4 | solute carrier family 15; member 4 | CCDS9264.1 | chr12_127859449-127859449__AGGG | NA | Insertion | Frameshift | GCTGCNGGGAG | 22% |
| LT-8 | SSPO | SCO-spondin precursor | NM_198455 | chr7_149130069-149130069_G_A | 2503R>H | Substitution | Nonsynonymous coding | ACTGCNTGCTG | 25% |
| LT-8 | TMEM2 | transmembrane protein 2 | CCDS6638.1 | chr9_73519731-73519731_G_T | 844Q>K | Substitution | Nonsynonymous coding | GTTCTNACCAC | 12% |
| LT-8 | TSPAN32 | tetraspanin 32 | NM_139022 | chr11_2295161-2295161_G_A | 244R>H | Substitution | Nonsynonymous coding | TGGCCNCCAGC | 24% |
| LT-8 | ZCCHC11 | zinc finger; CCHC domain containing 11 | CCDS30715.1 | chr1_52684686-52684686_G_A | 1177S>L | Substitution | Nonsynonymous coding | CAAGTNAAGGT | 14% |
| ST-1 | FUBP1 | far upstream element (FUSE) binding protein 1 | CCDS683.1 | chr1_78208215-78208215__T | NA | Insertion | Frameshift | AGGTCNTTTTT | 64% |
| ST-2 | NIPBL | Nipped-B homolog (Drosophila) | CCDS3920.1 | chr5_37031660-37031660_G_T | 1101E>X | Substitution | Nonsense | CTTTTNAATGT | 60% |
| ST-3 | AL691432.53 | cell division cycle 2-like 1 (PITSLRE proteins) isoform 3 | ENST00000404249 | chr1_1578672-1578672__A | NA | Insertion | Frameshift | TACTTNAAAAA | 13% |
| ST-3 | ARHGAP22 | Rho GTPase activating protein 22 | CCDS7227.1 | chr10_49337819-49337819_C_A | 191E>D | Substitution | Nonsynonymous coding | AGCCCNTCCTC | 31% |
| ST-3 | BAX | BCL2-associated X protein | CCDS12742.1 | chr19_54156690-54156690_G_A | 188W>X | Substitution | Nonsense | ATCTGNAAGAA | 16% |
| ST-3 | BOC | Boc homolog (mouse) | CCDS2971.1 | chr3_114452200-114452200_G_A | 69R>H | Substitution | Nonsynonymous coding | CTGGCNCCTGA | 12% |
| ST-3 | CALML3 | calmodulin-like 3 | CCDS7069.1 | chr10_5557298-5557298_G_A | 84E>K | Substitution | Nonsynonymous coding | ACGAGNAGGAG | 35% |
| ST-3 | CERKL | ceramide kinase-like | CCDS42789.1 | chr2_182177055-182177055__A | NA | Insertion | Splice site acceptor | ACCTGNAAAAA | 11% |
| ST-3 | CHST4 | carbohydrate (N-acetylglucosamine 6-O) sulfotransferase 4 | CCDS10902.1 | chr16_70128469-70128469_C_T | 130R>W | Substitution | Nonsynonymous coding | ACAGCNGGGCC | 12% |
| ST-3 | CYLC2 | cylicin; basic protein of sperm head cytoskeleton 2 | CCDS35085.1 | chr9_104806809-104806809_A_C | 64E>D | Substitution | Nonsynonymous coding | GATGANGAACA | 12% |
| ST-3 | DENND1A | DENN/MADD domain containing 1A | CCDS35133.1 | chr9_125242470-125242470_T_C | 493H>R | Substitution | Nonsynonymous coding | CAAAGNGGACT | 24% |
| ST-3 | DPH1 | DPH1 homolog (S. cerevisiae) | CCDS42228.1 | chr17_1890268-1890268_C_G | 257D>E | Substitution | Nonsynonymous coding | TATGANCCATA | 11% |
| ST-3 | EIF3L | eukaryotic translation initiation factor 3; subunit L | CCDS13960.1 | chr22_36612733-36612733_G_A | 531A>T | Substitution | Nonsynonymous coding | ACATCNCGGAC | 41% |
| ST-3 | ETFB | electron-transfer-flavoprotein; beta polypeptide | CCDS33085.1 | chr19_56542123-56542123_C_T | 238G>D | Substitution | Nonsynonymous coding | ATGTGNCCTGG | 21% |
| ST-3 | FAM98A | family with sequence similarity 98; member A | CCDS33179.1 | chr2_33665186-33665186_T_C | 223I>V | Substitution | Nonsynonymous coding | TTTTANTAGCA | 43% |
| ST-3 | GABRB2 | gamma-aminobutyric acid (GABA) A receptor; beta 2 | CCDS4355.1 | chr5_160690497-160690497_T_C | 350N>D | Substitution | Nonsynonymous coding | CTCATNGTTGG | 17% |
| ST-3 | HAUS4 | HAUS augmin-like complex; subunit 4 | CCDS9580.1 | chr14_22490741-22490742_GA_ | NA | Deletion | Splice site acceptor | AAACTNAGAGA | 11% |
| ST-3 | IDH1 | isocitrate dehydrogenase 1 (NADP+); soluble | CCDS2381.1 | chr2_208821357-208821357_C_T | 132R>H | Substitution | Nonsynonymous coding | CATGANGACCT | 35% |
| ST-3 | KIAA1949 | uncharacterized protein | CCDS43444.1 | chr6_30761488-30761488_T_C | 96Q>R | Substitution | Nonsynonymous coding | GCTGCNGCCGC | 39% |
| ST-3 | MEGF6 | multiple EGF-like-domains 6 | CCDS41237.1 | chr1_3411858-3411858_G_A | 681R>C | Substitution | Nonsynonymous coding | ACAGCNCTCGC | 15% |
| ST-3 | NCKAP1L | NCK-associated protein 1-like | CCDS31813.1 | chr12_53200787-53200787_G_C | 556L>F | Substitution | Nonsynonymous coding | ACCTTNGAGGA | 12% |
| ST-3 | NOTCH2 | Notch homolog 2 (Drosophila) | CCDS908.1 | chr1_120413526-120413527_GG_ | NA | Deletion | Frameshift | AGAGCNGGGCG | 29% |
| ST-3 | NPEPPS | aminopeptidase puromycin sensitive | NM_006310 | chr17_43024358-43024358_T_G | 433F>C | Substitution | Nonsynonymous coding | GATATNTGATG | 10% |
| ST-3 | PARP14 | poly [ADP-ribose] polymerase 14 | NM_017554 | chr3_123897175-123897175_C_G | 271L>V | Substitution | Nonsynonymous coding | CAGCTNTGATT | 14% |
| ST-3 | PCDHGA5 | protocadherin gamma-A5 isoform 1 precursor | NM_018918 | chr5_140725672-140725672_T_A | 531W>R | Substitution | Nonsynonymous coding | AGTTGNGGGTG | 14% |
| ST-3 | PRTN3 | proteinase 3 | CCDS32860.1 | chr19_794473-794473_C_T | 25A>V | Substitution | Nonsynonymous coding | CCGAGNTGCGG | 11% |
| ST-3 | PSENEN | presenilin enhancer 2 homolog (C. elegans) | CCDS12474.1 | chr19_40929163-40929163_G_A | 22G>E | Substitution | Nonsynonymous coding | AGGGGNGTTTG | 11% |
| ST-3 | RLBP1L2 | Retinaldehyde-binding protein 1-like protein 2. | CCDS34525.1 | chr6_123373852-123373852_G_A | 138R>H | Substitution | Nonsynonymous coding | TTTGCNTGCCA | 19% |
| ST-3 | RNF44 | ring finger protein 44 | CCDS4404.1 | chr5_175889128-175889128_A_C | NA | Substitution | Splice site donor | TACTCNCAGCG | 33% |
| ST-3 | RPRD2 | regulation of nuclear pre-mRNA domain containing 2 | NM_015203 | chr1_148710650-148710650_G_A | 868E>K | Substitution | Nonsynonymous coding | CCACCNAGTAC | 13% |
| ST-3 | SHANK1 | SH3 and multiple ankyrin repeat domains 1 | CCDS12799.1 | chr19_55862945-55862945_G_A | 1362R>X | Substitution | Nonsense | CGCTCNCTCGC | 15% |
| ST-3 | SHC1 | SHC (Src homology 2 domain containing) transforming protein 1 | CCDS30881.1 | chr1_153207698-153207698_A_G | 216L>P | Substitution | Nonsynonymous coding | AGCTGNGCGGG | 11% |
| ST-3 | SNAPC1 | small nuclear RNA activating complex; polypeptide 1; 43kDa | CCDS9755.1 | chr14_61303705-61303705_G_A | 104W>X | Substitution | Nonsense | GGATTNGGATG | 29% |
| ST-3 | SOX4 | SRY (sex determining region Y)-box 4 | CCDS4547.1 | chr6_21703122-21703122__C | NA | Insertion | Frameshift | GACTANAAGTA | 25% |
| ST-3 | TCHP | trichoplein; keratin filament binding | CCDS9137.1 | chr12_108836759-108836759_G_A | 422E>K | Substitution | Nonsynonymous coding | GTCGCNAGAAA | 38% |
| ST-3 | TPH2 | tryptophan hydroxylase 2 | ENST00000266669 | chr12_70641654-70641654_A_ | NA | Deletion | Frameshift | TCTGTNTCAAG | 24% |
| ST-3 | ZBTB20 | zinc finger and BTB domain containing 20 | CCDS2981.1 | chr3_115552881-115552881_G_A | 172S>L | Substitution | Nonsynonymous coding | GTGCCNAGTAG | 33% |
| ST-3 | ZFPM2 | zinc finger protein; multitype 2 | NM_012082 | chr8_106882741-106882741_G_A | 419E>K | Substitution | Nonsynonymous coding | GAAGCNAACTT | 10% |
| ST-4 | ABHD6 | abhydrolase domain containing 6 | CCDS2887.1 | chr3_58217375-58217375_A_G | 8M>V | Substitution | Nonsynonymous coding | TTAACNTGTTT | 40% |
| ST-4 | ACIN1 | apoptotic chromatin condensation inducer 1 | CCDS9587.1 | chr14_22600415-22600415_C_ | NA | Deletion | Frameshift | TTGATNGGGAA | 32% |
| ST-4 | ADAM2 | ADAM metallopeptidase domain 2 | CCDS34884.1 | chr8_39797769-39797769_T_C | 141Y>C | Substitution | Nonsynonymous coding | CTTGGNAAATT | 30% |
| ST-4 | ADNP | activity-dependent neuroprotector homeobox | CCDS13433.1 | chr20_48941849-48941849_C_T | 937E>K | Substitution | Nonsynonymous coding | AGTTTNGTATT | 34% |
| ST-4 | ANKRD26 | ankyrin repeat domain 26 | CCDS41499.1 | chr10_27364167-27364167_A_C | 1073L>R | Substitution | Nonsynonymous coding | TATTGNGTTTA | 32% |
| ST-4 | CIC | capicua homolog (Drosophila) | CCDS12601.1 | chr19_47482781-47482784_CTCG_ | NA | Deletion | Frameshift | GGAACNTCGCT | 42% |
| ST-4 | EGFL8 | epidermal growth factor 8lysosomal thioesterase PPT2 | CCDS4743.1 | chr6_32243701-32243701_A_G | 291N>S | Substitution | Nonsynonymous coding | CGTCANTCATC | 33% |
| ST-4 | EPX | eosinophil peroxidase | CCDS11602.1 | chr17_53631547-53631547_G_A | 423G>E | Substitution | Nonsynonymous coding | CATGGNGGCCA | 37% |
| ST-4 | FLT1 | fms-related tyrosine kinase 1 (vascular endothelial growth factor/vascular permeability factor receptor) | CCDS9330.1 | chr13_27862191-27862191_C_T | 571E>K | Substitution | Nonsynonymous coding | GTCCTNTCCTT | 12% |
| ST-4 | HPS1 | Hermansky-Pudlak syndrome 1 | CCDS7475.1 | chr10_100175342-100175342_G_C | 427D>E | Substitution | Nonsynonymous coding | CGCAGNTCTCC | 29% |
| ST-4 | HSPG2 | heparan sulfate proteoglycan 2 | CCDS30625.1 | chr1_22030097-22030097_C_T | 3879R>H | Substitution | Nonsynonymous coding | CACAGNGGCTC | 53% |
| ST-4 | IDH1 | isocitrate dehydrogenase 1 (NADP+); soluble | CCDS2381.1 | chr2_208821357-208821357_C_T | 132R>H | Substitution | Nonsynonymous coding | CATGANGACCT | 29% |
| ST-4 | IGHV3-64 | Immunoglobulin heavy chain V gene segment | ENST00000390631 | chr14_106184910-106184910_C_A | 77S>I | Substitution | Nonsynonymous coding | ATGTGNTACCC | 12% |
| ST-4 | ITGA5 | integrin; alpha 5 (fibronectin receptor; alpha polypeptide) | CCDS8880.1 | chr12_53083780-53083780_G_A | 558R>W | Substitution | Nonsynonymous coding | CCGCCNTACCC | 25% |
| ST-4 | PDF | peptide deformylase (mitochondrial) | CCDS10876.1 | chr16_67922299-67922299_A_T | 595C>S | Substitution | Nonsynonymous coding | CGGGCNGGCTG | 34% |
| ST-4 | PIK3CA | phosphoinositide-3-kinase; catalytic; alpha polypeptide | CCDS43171.1 | chr3_180434779-180434779_A_G | 1047H>R | Substitution | Nonsynonymous coding | TGCACNTCATG | 28% |
| ST-4 | PRG4 | proteoglycan 4 | CCDS1369.1 | chr1_184543191-184543191_C_A | 573P>T | Substitution | Nonsynonymous coding | CCACCNCCAAG | 13% |
| ST-4 | RIF1 | RAP1 interacting factor homolog (yeast) | CCDS2194.1 | chr2_151985143-151985143_A_G | NA | Substitution | Splice site donor | CTGTGNGTGTT | 26% |
| ST-4 | RNF6 | ring finger protein (C3H2C3 type) 6 | CCDS9316.1 | chr13_25687361-25687361_T_C | 220R>G | Substitution | Nonsynonymous coding | CCCCCNTGAAG | 29% |
| ST-4 | RUFY2 | RUN and FYVE domain containing 2 | CCDS41534.1 | chr10_69826589-69826589_T_ | NA | Deletion | Frameshift | GGCCANTTTTT | 17% |
| ST-4 | SNTG1 | syntrophin; gamma 1 | CCDS6147.1 | chr8_51867917-51867917_C_T | 510T>M | Substitution | Nonsynonymous coding | TACCANGAGCA | 15% |
| ST-4 | TMEM135 | transmembrane protein 135 | CCDS8280.1 | chr11_86546086-86546086_G_T | 151R>I | Substitution | Nonsynonymous coding | ATTAANAAATG | 27% |
| ST-4 | TTN | titin | ENST00000375038 | chr2_179177958-179177958_C_T | NA | Substitution | Splice site donor | ACTTANCATAT | 30% |
| ST-5 | ABP1 | amiloride binding protein 1 (amine oxidase (copper-containing)) | CCDS43679.1 | chr7_150184685-150184685_C_T | 65A>V | Substitution | Nonsynonymous coding | CATGGNCAAGA | 39% |
| ST-5 | ADAM8 | ADAM metallopeptidase domain 8 | CCDS31319.1 | chr10_134931460-134931460_G_A | 739P>L | Substitution | Nonsynonymous coding | AGGCCNGGTGT | 25% |
| ST-5 | ADAMTS20 | A disintegrin and metalloproteinase with thrombospondin motifs 20 preproprotein | NM_025003 | chr12_42109741-42109741_T_G | 1145L>F | Substitution | Nonsynonymous coding | GTTGGNAATAA | 16% |
| ST-5 | ARMC6 | armadillo repeat containing 6 | CCDS32965.1 | chr19_19027137-19027137_G_A | 338A>T | Substitution | Nonsynonymous coding | AAGATNCTATT | 11% |
| ST-5 | C10orf79 | chromosome 10 open reading frame 79 | CCDS31281.1 | chr10_105980403-105980403_C_T | 85R>Q | Substitution | Nonsynonymous coding | GCTTCNGGTCA | 37% |
| ST-5 | C14orf115 | chromosome 14 open reading frame 115 | CCDS9830.1 | chr14_73893796-73893796_A_G | 186N>S | Substitution | Nonsynonymous coding | GCGGANCATCT | 72% |
| ST-5 | CDC42EP4 | CDC42 effector protein (Rho GTPase binding) 4 | CCDS11695.1 | chr17_68793627-68793627_G_A | 203A>V | Substitution | Nonsynonymous coding | ACTCCNCATGC | 14% |
| ST-5 | CNIH | cornichon homolog (Drosophila) | CCDS9717.1 | chr14_53968646-53968646_T_C | 65M>V | Substitution | Nonsynonymous coding | AAACANGACAC | 31% |
| ST-5 | CTU1 | cytosolic thiouridylase subunit 1 homolog (S. pombe) | CCDS12824.1 | chr19_56299370-56299370_C_G | 90G>A | Substitution | Nonsynonymous coding | CGATGNCCTCA | 14% |
| ST-5 | EML1 | echinoderm microtubule associated protein like 1 | CCDS32154.1 | chr14_99434388-99434388_G_A | 317G>E | Substitution | Nonsynonymous coding | GGATGNAAAAG | 80% |
| ST-5 | EML6 | echinoderm microtubule-associated protein-like 6 | NM_001039753 | chr2_54901052-54901052_G_A | 158W>X | Substitution | Nonsense | TCCTGNGATCC | 38% |
| ST-5 | EP300 | E1A binding protein p300 | CCDS14010.1 | chr22_39886673-39886676_GTAA_ | NA | Deletion | Splice site donor | CAAACNTAAGT | 22% |
| ST-5 | FMO4 | flavin containing monooxygenase 4 | CCDS1295.1 | chr1_169567537-169567537_G_A | 207A>T | Substitution | Nonsynonymous coding | GAACGNCAGCT | 34% |
| ST-5 | FUBP1 | far upstream element (FUSE) binding protein 1 | CCDS683.1 | chr1_78206436-78206439_CAGT_ | NA | Deletion | Splice site donor | ACTTANAGTCA | 50% |
| ST-5 | GGH | gamma-glutamyl hydrolase (conjugase; folylpolygammaglutamyl hydrolase) | CCDS6177.1 | chr8_64110838-64110838_C_G | 52G>A | Substitution | Nonsynonymous coding | ATCTTNCATAG | 25% |
| ST-5 | HNRNPA1 | heterogeneous nuclear ribonucleoprotein A1 | CCDS41793.1 | chr12_52962532-52962533_AA_ | NA | Deletion | Frameshift | CAGCCNAAGAG | 30% |
| ST-5 | HTR6 | 5-hydroxytryptamine (serotonin) receptor 6 | CCDS197.1 | chr1_19878287-19878287_G_A | 388G>S | Substitution | Nonsynonymous coding | CAGGCNGCTCC | 20% |
| ST-5 | IDH1 | isocitrate dehydrogenase 1 (NADP+); soluble | CCDS2381.1 | chr2_208821357-208821357_C_T | 132R>H | Substitution | Nonsynonymous coding | CATGANGACCT | 35% |
| ST-5 | INO80B | INO80 complex subunit B | CCDS1942.2 | chr2_74536816-74536817_AG_ | NA | Deletion | Frameshift | GGAACNGAGGT | 20% |
| ST-5 | MGA | MAX gene-associated protein isoform 1 | NM_001164273 | chr15_39830013-39830013_G_A | 2306E>K | Substitution | Nonsynonymous coding | ATGATNAAGAT | 37% |
| ST-5 | MPPED2 | metallophosphoesterase domain containing 2 | CCDS7870.1 | chr11_30392369-30392369_A_G | 250F>L | Substitution | Nonsynonymous coding | ACCAANCACAT | 25% |
| ST-5 | MS4A1 | membrane-spanning 4-domains; subfamily A; member 1 | CCDS31570.1 | chr11_59992341-59992341_A_G | 240I>V | Substitution | Nonsynonymous coding | AGACTNTTGAA | 39% |
| ST-5 | NFIB | nuclear factor I/B | CCDS6474.1 | chr9_14303479-14303479_A_G | NA | Substitution | Splice site donor | CATGTNCCTGA | 11% |
| ST-5 | PDE4B | phosphodiesterase 4B; cAMP-specific (phosphodiesterase E4 dunce homolog; Drosophila) | CCDS632.1 | chr1_66600045-66600045_A_G | 334N>S | Substitution | Nonsynonymous coding | TGAAANTGAAG | 24% |
| ST-5 | POU4F3 | POU class 4 homeobox 3 | CCDS4281.1 | chr5_145699460-145699460_T_C | 93S>P | Substitution | Nonsynonymous coding | CTTCGNCCACC | 17% |
| ST-5 | PTPRB | receptor-type tyrosine-protein phosphatase beta isoform a | NM_001109754 | chr12_69246502-69246502_G_C | 1295A>G | Substitution | Nonsynonymous coding | TCTGGNCTTCC | 46% |
| ST-5 | PXDNL | peroxidasin-like protein precursor | NM_144651 | chr8_52528847-52528847_G_C | 345T>S | Substitution | Nonsynonymous coding | TGCTGNTGCCA | 16% |
| ST-5 | PYGB | phosphorylase; glycogen; brain | CCDS13171.1 | chr20_25177044-25177044_A_G | 77E>G | Substitution | Nonsynonymous coding | CTACGNGCGCG | 32% |
| ST-5 | SCD5 | stearoyl-CoA desaturase 5 | CCDS34024.1 | chr4_83821032-83821032_C_T | 141D>N | Substitution | Nonsynonymous coding | AGCATNCGTCT | 17% |
| ST-5 | SLITRK6 | SLIT and NTRK-like family; member 6 | CCDS41903.1 | chr13_85267693-85267693_T_C | 318T>A | Substitution | Nonsynonymous coding | CTTTGNAATAT | 40% |
| ST-5 | SMURF1 | SMAD specific E3 ubiquitin protein ligase 1 | CCDS34690.1 | chr7_98481351-98481351_G_A | 414R>C | Substitution | Nonsynonymous coding | CTGGCNGTAAG | 42% |
| ST-5 | SPERT | spermatid associated | CCDS9399.1 | chr13_45185580-45185580_G_T | 140C>F | Substitution | Nonsynonymous coding | GAACTNCCGCC | 37% |
| ST-5 | TFPT | TCF3 (E2A) fusion partner (in childhood Leukemia) | CCDS12878.1 | chr19_59303300-59303303_TCTC_ | NA | Deletion | Frameshift | CTCTTNCTCTG | 20% |
| ST-5 | TJP2 | tight junction protein 2 (zona occludens 2) | CCDS6627.1 | chr9_71052925-71052925_A_G | 949I>V | Substitution | Nonsynonymous coding | CGTCCNTCACC | 12% |
| ST-5 | TTC29 | tetratricopeptide repeat domain 29 | NM_031956 | chr4_148080437-148080437_T_C | 21K>E | Substitution | Nonsynonymous coding | CAGCTNCTGTC | 14% |
| ST-5 | TTC8 | tetratricopeptide repeat domain 8 | CCDS32137.1 | chr14_88408493-88408493_G_A | 431A>T | Substitution | Nonsynonymous coding | ACCACNCCGAG | 14% |
| ST-5 | TTN | titin | ENST00000375038 | chr2_179106416-179106416_G_ | NA | Deletion | Frameshift | TTGGTNGGGGG | 31% |
| ST-6 | ABCF1 | ATP-binding cassette, sub-family F (GCN20), member 1 | CCDS34380.1 | chr6_30666457-30666457__A | NA | Insertion | Frameshift | GAGTGNGCTTT | 12% |
| ST-6 | ADAMTS1 | ADAM metallopeptidase with thrombospondin type 1 motif, 1 | ENST00000382884 | chr21_27137930-27137930_A_G | NA | Substitution | Splice site donor | AGGGTNCCTTC | 10% |
| ST-6 | ADAMTSL4 | ADAMTS-like 4 | CCDS955.1 | chr1_148798462-148798462_G_A | 947V>M | Substitution | Nonsynonymous coding | TCAACNTGACT | 10% |
| ST-6 | ADCY3 | adenylate cyclase 3 | CCDS1715.1 | chr2_24914819-24914819_G_A | 511S>L | Substitution | Nonsynonymous coding | TCACCNAGCCA | 18% |
| ST-6 | ADH4 | alcohol dehydrogenase 4 (class II), pi polypeptide | CCDS34032.1 | chr4_100266767-100266767_C_T | NA | Substitution | Splice site donor | ATGTANCTTTT | 10% |
| ST-6 | ASPDH | aspartate dehydrogenase domain containing | CCDS33082.1 | chr19_55707216-55707216_T_C | 161Q>R | Substitution | Nonsynonymous coding | GGCTCNGCCAG | 11% |
| ST-6 | AVEN | apoptosis, caspase activation inhibitor | CCDS10030.1 | chr15_31947315-31947315_G_A | 216P>S | Substitution | Nonsynonymous coding | CTTTGNTTTCA | 35% |
| ST-6 | AVIL | advillin | CCDS8959.1 | chr12_56487772-56487772_C_G | 400W>C | Substitution | Nonsynonymous coding | ATTCTNCAGAC | 11% |
| ST-6 | BASH | B-cell linker | ENST00000393898 | chr10_97949227-97949227_C_A | NA | Substitution | Splice site donor | ACCTCNCCTGG | 11% |
| ST-6 | BEND6 | BEN domain containing 6 | CCDS43476.1 | chr6_56965268-56965268_G_A | 85R>Q | Substitution | Nonsynonymous coding | CACCCNGAAAG | 13% |
| ST-6 | BHLHE22 | basic helix-loop-helix family, member e22 | CCDS6179.1 | chr8_65656086-65656086_T_A | 62L>Q | Substitution | Nonsynonymous coding | GCCCCNGGGCT | 11% |
| ST-6 | BMP1 | bone morphogenetic protein 1 | ENST00000354870 | chr8_22091194-22091194__G | NA | Insertion | Splice site donor | AGGTANGGGGA | 14% |
| ST-6 | BTBD11 | BTB (POZ) domain containing 11 | CCDS41827.1 | chr12_106498874-106498874_G_A | 21G>R | Substitution | Nonsynonymous coding | GGTCCNGATGT | 14% |
| ST-6 | C1orf167 | chromosome 1 open reading frame 167 | ENST00000312793 | chr1_11762553-11762553_G_A | 131R>H | Substitution | Nonsynonymous coding | GTGGCNTCAGC | 11% |
| ST-6 | C7orf27 | BRCA1-associated ATM activator 1 | ENST00000382467 | chr7_2548520-2548520_T_C | 226I>V | Substitution | Nonsynonymous coding | GGGGANGCCAT | 15% |
| ST-6 | C7orf27 | BRCA1-associated ATM activator 1 | ENST00000382467 | chr7_2548759-2548759_A_G | 146I>T | Substitution | Nonsynonymous coding | CCACANTGCAT | 13% |
| ST-6 | C9orf144A | family with sequence similarity 205, member B | ENST00000291050 | chr9_34824134-34824134_A_G | 452L>P | Substitution | Nonsynonymous coding | GTACCNGGAAC | 12% |
| ST-6 | CAPN3 | calpain 3, (p94) | CCDS32207.1 | chr15_40489335-40489335_G_A | NA | Substitution | Splice site donor | CAAACNTGAGT | 11% |
| ST-6 | CASP8AP2 | caspase 8 associated protein 2 | NM_001137667 | chr6_90637739-90637739_C_T | 1935R>W | Substitution | Nonsynonymous coding | ATGACNGGGAA | 12% |
| ST-6 | CCDC108 | coiled-coil domain containing 108 | CCDS2430.2 | chr2_219603792-219603792_C_A | 342A>S | Substitution | Nonsynonymous coding | CTGGGNGCACT | 14% |
| ST-6 | CCDC155 | coiled-coil domain containing 155 | NM_144688 | chr19_54590216-54590216_G_A | 64V>M | Substitution | Nonsynonymous coding | AGGCTNTGACA | 29% |
| ST-6 | CCRL2 | chemokine (C-C motif) receptor-like 2 | CCDS43079.1 | chr3_46424867-46424867_G_A | 98G>E | Substitution | Nonsynonymous coding | TGCTGNGGGCG | 12% |
| ST-6 | CDK10 | cyclin-dependent kinase 10 | ENST00000378277 | chr16_88288213-88288213_G_A | 182E>K | Substitution | Nonsynonymous coding | TTCCTNAGCTC | 13% |
| ST-6 | CHGB | chromogranin B (secretogranin 1) | CCDS13092.1 | chr20_5851067-5851067_T_A | 93S>T | Substitution | Nonsynonymous coding | ATGCCNCGGAA | 10% |
| ST-6 | CNGA2 | cyclic nucleotide gated channel alpha 2 | CCDS14701.1 | chrX_150659963-150659963_G_T | 139W>L | Substitution | Nonsynonymous coding | GGATTNGTACT | 11% |
| ST-6 | CNTN5 | contactin 5 | NM_001243270 | chr11_99195496-99195496_T_G | 23S>A | Substitution | Nonsynonymous coding | CAAAANCTCTT | 16% |
| ST-6 | CYP2F1 | cytochrome P450, family 2, subfamily F, polypeptide 1 | CCDS12572.1 | chr19_46313948-46313948__C | NA | Insertion | Frameshift | ATAAGNACAGC | 23% |
| ST-6 | DCHS2 | dachsous cadherin-related 2 | NM_001142552 | chr4_155630100-155630100_T_C | 620T>A | Substitution | Nonsynonymous coding | TAGAGNCCGGA | 13% |
| ST-6 | DCHS2 | dachsous cadherin-related 2 | NM_001142552 | chr4_155630117-155630117_G_A | 614A>V | Substitution | Nonsynonymous coding | TGATCNCACCG | 13% |
| ST-6 | DEPDC5 | DEP domain containing 5 | CCDS43006.1 | chr22_30548727-30548727_C_A | 685F>L | Substitution | Nonsynonymous coding | AACTTNAGTGG | 13% |
| ST-6 | DNAJC25 | DnaJ (Hsp40) homolog, subfamily C , member 25 | CCDS43862.1 | chr9_113433605-113433605_G_A | 33V>M | Substitution | Nonsynonymous coding | TGCTGNTGCGG | 14% |
| ST-6 | EFCAB4A | EF-hand calcium binding domain 4A | CCDS41588.1 | chr11_819539-819539_A_G | 153K>E | Substitution | Nonsynonymous coding | TGGGCNAGTGA | 25% |
| ST-6 | EIF4B | eukaryotic translation initiation factor 4B | CCDS41788.1 | chr12_51701906-51701906_A_G | 176D>G | Substitution | Nonsynonymous coding | ACAGGNTAAAG | 40% |
| ST-6 | FBXO10 | F-box protein 10 | NM_012166 | chr9_37512963-37512963_G_A | 597R>C | Substitution | Nonsynonymous coding | CTCACNGATGA | 35% |
| ST-6 | FCGBP | Fc fragment of IgG binding protein | CCDS12546.1 | chr19_45125325-45125325_G_A | 262R>C | Substitution | Nonsynonymous coding | ATAGCNAGATT | 10% |
| ST-6 | FDPS | farnesyl diphosphate synthase | CCDS1110.1 | chr1_153556855-153556855_T_C | 364V>A | Substitution | Nonsynonymous coding | GAAAGNGGCCC | 17% |
| ST-6 | FREM1 | FRAS1 related extracellular matrix 1 | NM_144966 | chr9_14736936-14736936_C_T | 2041W>X | Substitution | Nonsense | GGACTNCAGGC | 37% |
| ST-6 | FUBP1 | far upstream element (FUSE) binding protein 1 | CCDS683.1 | chr1_78208234-78208234_A_C | 58Y>X | Substitution | Nonsense | TAACCNTAGTC | 74% |
| ST-6 | GALNT16 | UDP-N-acetyl-alpha-D-galactosamine:polypeptide N-acetylgalactosaminyltransferase 16 | CCDS32107.1 | chr14_68862480-68862480_G_A | 184R>H | Substitution | Nonsynonymous coding | CCTGCNCAATG | 15% |
| ST-6 | GH1 | growth hormone 1 | CCDS11653.1 | chr17_59348917-59348917_C_T | 131A>T | Substitution | Nonsynonymous coding | AGAGGNGCCGT | 14% |
| ST-6 | GLIPR1L2 | GLI pathogenesis-related 1 like 2 | CCDS9010.1 | chr12_74103082-74103082__ACA | 239D>DN | Insertion | In-frame insertion | ATTGGNCAAGA | 13% |
| ST-6 | HCRTR1 | hypocretin (orexin) receptor 1 | CCDS344.1 | chr1_31857797-31857797_G_A | 93V>I | Substitution | Nonsynonymous coding | CTGACNTTCTG | 11% |
| ST-6 | HSP90B1 | heat shock protein 90kDa beta (Grp94), member 1 | CCDS9094.1 | chr12_102856354-102856354_C_T | 321P>L | Substitution | Nonsynonymous coding | GAAACNAAAGA | 11% |
| ST-6 | HTR3D | 5-hydroxytryptamine (serotonin) receptor 3D, ionotropic | CCDS3249.1 | chr3_185239396-185239396_G_A | 260R>H | Substitution | Nonsynonymous coding | CTTCCNCCTCT | 10% |
| ST-6 | IGDCC4 | immunoglobulin superfamily, DCC subclass, member 4 | CCDS10206.1 | chr15_63490678-63490678_C_G | 52A>P | Substitution | Nonsynonymous coding | TGCAGNCTGCT | 11% |
| ST-6 | KIF12 | kinesin family member 12 | CCDS6801.1 | chr9_115893967-115893967_G_A | 513H>Y | Substitution | Nonsynonymous coding | TCAATNGGGAG | 11% |
| ST-6 | LAMTOR5 | late endosomal/lysosomal adaptor, MAPK and MTOR activator 5 | CCDS824.1 | chr1_110751730-110751730_C_T | NA | Substitution | Splice site donor | ACTCANGTGTC | 21% |
| ST-6 | LRRC26 | leucine rich repeat containing 26 | CCDS35184.1 | chr9_139184136-139184136_C_A | 27Q>H | Substitution | Nonsynonymous coding | GACACNTGGGC | 10% |
| ST-6 | MAD2L2 | MAD2 mitotic arrest deficient-like 2 (yeast) | ENST00000376655 | chr1_11660138-11660138_C_T | 94V>I | Substitution | Nonsynonymous coding | AGGGANGGGGG | 13% |
| ST-6 | MAST4 | microtubule associated serine/threonine kinase family member 4 | NM_001164664 | chr5_65928524-65928524__GCC | 95L>LP | Insertion | In-frame insertion | GCGCTNCCGCC | 22% |
| ST-6 | MIB2 | mindbomb E3 ubiquitin protein ligase 2 | CCDS41224.1 | chr1_1553307-1553307_A_G | 634T>A | Substitution | Nonsynonymous coding | CGGGCNCTGGA | 10% |
| ST-6 | MORC4 | MORC family CW-type zinc finger 4 | CCDS14525.2 | chrX_106115007-106115007_G_A | 217R>C | Substitution | Nonsynonymous coding | AACACNAGTGC | 13% |
| ST-6 | MS4A15 | membrane-spanning 4-domains, subfamily A, member 15 | NM_001098835 | chr11_60287840-60287840_A_G | 20S>G | Substitution | Nonsynonymous coding | ACGCCNGTGGC | 11% |
| ST-6 | MTCH1 | mitochondrial carrier 1 | ENST00000337855 | chr6_37044618-37044618_G_A | 321P>L | Substitution | Nonsynonymous coding | CCCACNGTGGC | 10% |
| ST-6 | NLRP8 | NLR family, pyrin domain containing 8 | CCDS12937.1 | chr19_61159157-61159157_G_T | 641E>X | Substitution | Nonsense | ACAAANAAGTT | 12% |
| ST-6 | NOTCH1 | notch 1 | CCDS43905.1 | chr9_138519187-138519187_G_ | NA | Deletion | Frameshift | CCGCANGAAGT | 67% |
| ST-6 | PAPLN | papilin, proteoglycan-like sulfated glycoprotein | CCDS32114.1 | chr14_72797716-72797716_G_A | 709G>S | Substitution | Nonsynonymous coding | CAGCCNGTCCT | 12% |
| ST-6 | PIGT | phosphatidylinositol glycan anchor biosynthesis, class T | CCDS13353.1 | chr20_43483470-43483470_G_A | 356R>Q | Substitution | Nonsynonymous coding | CCAGCNGTACG | 10% |
| ST-6 | PLCH2 | phospholipase C, eta 2 | ENST00000343889 | chr1_2423760-2423760_C_T | 851P>L | Substitution | Nonsynonymous coding | GCCCCNGCGTG | 12% |
| ST-6 | PLCH2 | phospholipase C, eta 2 | NM_014638 | chr1_2426264-2426264_G_C | 1335V>L | Substitution | Nonsynonymous coding | GTTTTNTGCGG | 11% |
| ST-6 | PMS2 | PMS2 postmeiotic segregation increased 2 (S. cerevisiae) | CCDS5343.1 | chr7_5993468-5993468_G_T | 485T>K | Substitution | Nonsynonymous coding | TGTCCNTAGGG | 12% |
| ST-6 | PTPN7 | protein tyrosine phosphatase, non-receptor type 7 | NM_002832 | chr1_200396518-200396518_G_A | 14T>M | Substitution | Nonsynonymous coding | GCCCCNTTCCC | 10% |
| ST-6 | RBPMS2 | RNA binding protein with multiple splicing 2 | CCDS32271.1 | chr15_62829632-62829632_C_T | 56G>E | Substitution | Nonsynonymous coding | CATACNCCTGC | 11% |
| ST-6 | RP1L1 | retinitis pigmentosa 1-like 1 | CCDS43708.1 | chr8_10502165-10502165_C_T | 2285G>R | Substitution | Nonsynonymous coding | GTCTCNACCTG | 12% |
| ST-6 | RP1L1 | retinitis pigmentosa 1-like 1 | CCDS43708.1 | chr8_10503352-10503352_T_A | 1889D>V | Substitution | Nonsynonymous coding | CTACANCTTCT | 11% |
| ST-6 | RP1L1 | retinitis pigmentosa 1-like 1 | CCDS43708.1 | chr8_10504534-10504534_G_C | 1495P>R | Substitution | Nonsynonymous coding | GGGTGNGTTGG | 12% |
| ST-6 | RPS21 | ribosomal protein S21 | ENST00000370592 | chr20_60396450-60396450_T_C | 91*>Q | Substitution | Nonsynonymous coding | CTGAGNAGATC | 10% |
| ST-6 | RPS6KA2 | ribosomal protein S6 kinase, 90kDa, polypeptide 2 | CCDS34570.1 | chr6_167191706-167191706_T_C | 32E>G | Substitution | Nonsynonymous coding | TGGGCNCCACA | 12% |
| ST-6 | SBSN | suprabasin | NM_001166034 | chr19_40709768-40709768_G_C | 419A>G | Substitution | Nonsynonymous coding | GCCCCNCCTCC | 15% |
| ST-6 | SCN10A | sodium channel, voltage-gated, type X, alpha subunit | CCDS33736.1 | chr3_38780056-38780057_GA_ | NA | Deletion | Frameshift | GGCCTNAGATC | 49% |
| ST-6 | SERPINA4 | serpin peptidase inhibitor, clade A (alpha-1 antiproteinase, antitrypsin), member 4 | CCDS9927.1 | chr14_94100185-94100185_G_A | 205V>I | Substitution | Nonsynonymous coding | AGGACNTCTTG | 37% |
| ST-6 | SFTPB | surfactant protein B | NM_000542 | chr2_85748849-85748849_T_G | 2H>P | Substitution | Nonsynonymous coding | CTTGGNGCATG | 10% |
| ST-6 | SGK223 | tyrosine-protein kinase SgK223 | CCDS43706.1 | chr8_8272124-8272124_C_T | 402R>Q | Substitution | Nonsynonymous coding | CCTCCNGGGGG | 13% |
| ST-6 | SGSM3 | small G protein signaling modulator 3 | CCDS14002.1 | chr22_39135321-39135321_C_T | 704R>X | Substitution | Nonsense | AGCTCNGGTGA | 11% |
| ST-6 | SMYD3 | SET and MYND domain containing 3 | CCDS31083.1 | chr1_244557215-244557215_C_T | 89V>I | Substitution | Nonsynonymous coding | CATTANGAGTT | 47% |
| ST-6 | SRRM1 | serine/arginine repetitive matrix 1 | CCDS255.1 | chr1_24850561-24850561_G_A | 199R>Q | Substitution | Nonsynonymous coding | TTCTCNATCTC | 11% |
| ST-6 | SSC5D | scavenger receptor cysteine rich domain containing (5 domains) | NM_001144950 | chr19_60701277-60701277_G_A | 536G>S | Substitution | Nonsynonymous coding | ATGTGNGCTGT | 11% |
| ST-6 | SUPT20HL2 | suppressor of Ty 20 homolog (S. cerevisiae)-like 2 | NM_001136233 | chrX_24239970-24239970_G_A | 462P>S | Substitution | Nonsynonymous coding | GGGAGNTGGAT | 11% |
| ST-6 | TAB1 | TGF-beta activated kinase 1/MAP3K7 binding protein 1 | CCDS13993.1 | chr22_38143746-38143746_G_A | 166A>T | Substitution | Nonsynonymous coding | GAGGGNCCATG | 15% |
| ST-6 | TAF1C | TATA box binding protein (TBP)-associated factor, RNA polymerase I, C, 110kDa | CCDS32496.1 | chr16_82770280-82770280_G_A | 793P>L | Substitution | Nonsynonymous coding | AGGGCNGGGTC | 16% |
| ST-6 | TCERG1L | transcription elongation regulator 1-like | NM_174937 | chr10_132822633-132822633_G_A | 420R>W | Substitution | Nonsynonymous coding | CTACCNGTTCC | 10% |
| ST-6 | TEP1 | telomerase-associated protein 1 | CCDS9548.1 | chr14_19933506-19933506_C_T | 624R>K | Substitution | Nonsynonymous coding | GTATCNTCATT | 12% |
| ST-6 | TMEM30B | transmembrane protein 30B | CCDS32093.1 | chr14_60817347-60817347_G_A | 91P>L | Substitution | Nonsynonymous coding | AGCAGNGGGGC | 10% |
| ST-6 | TNS1 | tensin 1 | CCDS2407.1 | chr2_218453962-218453962_C_G | 320G>R | Substitution | Nonsynonymous coding | CGGCCNGTTCT | 17% |
| ST-6 | TRPV2 | transient receptor potential cation channel, subfamily V, member 2 | CCDS32576.1 | chr17_16276211-16276211_A_T | 621M>L | Substitution | Nonsynonymous coding | GCGGCNTGGTG | 46% |
| ST-6 | TTBK1 | tau tubulin kinase 1 | CCDS34455.1 | chr6_43360911-43360911_C_G | 1262P>R | Substitution | Nonsynonymous coding | CTCCCNCTCGC | 12% |
| ST-6 | TTC3 | tetratricopeptide repeat domain 3 | CCDS13651.1 | chr21_37492172-37492172_A_C | 1942K>N | Substitution | Nonsynonymous coding | CAGAANGCAGA | 11% |
| ST-6 | TTLL10 | tubulin tyrosine ligase-like family, member 10 | NM_001130045 | chr1_1122936-1122936_G_A | 623R>Q | Substitution | Nonsynonymous coding | TCCCCNGCCAC | 13% |
| ST-6 | TTLL8 | tubulin tyrosine ligase-like family, member 8 | ENST00000266182 | chr22_48811034-48811034_C_T | 644G>S | Substitution | Nonsynonymous coding | CAGGCNCCGCA | 28% |
| ST-6 | UMODL1 | uromodulin-like 1 | CCDS42935.1 | chr21_42404077-42404077_T_C | 559M>T | Substitution | Nonsynonymous coding | CCCCANGGGCG | 14% |
| ST-6 | VAV1 | vav 1 guanine nucleotide exchange factor | CCDS12174.1 | chr19_6801767-6801767_C_T | 739T>M | Substitution | Nonsynonymous coding | GCTTANGGTAA | 11% |
| ST-6 | VIP | vasoactive intestinal peptide | CCDS5240.1 | chr6_153119093-153119093_G_A | 156S>N | Substitution | Nonsynonymous coding | GAGGANGTAAA | 21% |
| ST-6 | VSTM2A | V-set and transmembrane domain containing 2A | ENST00000404951 | chr7_54589186-54589186_G_A | 256R>Q | Substitution | Nonsynonymous coding | GAAGCNGCTTC | 11% |
| ST-6 | VWF | von Willebrand factor | ENST00000321023 | chr12_6074876-6074876_T_G | 260D>A | Substitution | Nonsynonymous coding | GCCAGNCCACA | 12% |
| ST-6 | WDR66 | WD repeat domain 66 | CCDS41853.1 | chr12_120880778-120880778_C_T | 650L>F | Substitution | Nonsynonymous coding | AGGGGNTTGGA | 16% |
| ST-6 | YLPM1 | YLP motif containing 1 | NM_019589 | chr14_74335984-74335984_G_C | 1411D>H | Substitution | Nonsynonymous coding | ATGTGNATAGA | 14% |
| ST-6 | ZNF350 | zinc finger protein 350 | CCDS12845.1 | chr19_57160218-57160218_C_A | 434V>L | Substitution | Nonsynonymous coding | TTCCANCTTGG | 13% |
| ST-6 | ZNF880 | zinc finger protein 880 | NM_001145434 | chr19_57580057-57580057_A_G | 471K>R | Substitution | Nonsynonymous coding | TGGCANGGACT | 12% |
| ST-7 | ARL13A | ADP-ribosylation factor-like 13A | NM_001162491 | chrX_100129098-100129098_C_T | 184R>C | Substitution | Nonsynonymous coding | GACTGNGCTGG | 11% |
| ST-7 | ASIC3 | acid-sensing (proton-gated) ion channel 3 | CCDS5915.1 | chr7_150379221-150379221_T_A | 366C>S | Substitution | Nonsynonymous coding | GCGCCNGCCCC | 38% |
| ST-7 | ASIC3 | acid-sensing (proton-gated) ion channel 3 | CCDS5915.1 | chr7_150379221-150379226_TGCCCC_AGCA | NA | Complex indel | Frameshift | GCCTGNCCCAA | 38% |
| ST-7 | CAMSAP1 | calmodulin regulated spectrin-associated protein 1 | CCDS35176.1 | chr9_137855621-137855621__T | 562dupA | Insertion | Frameshift | CCTGGNTTTTT | 11% |
| ST-7 | CCDC154 | coiled-coil domain containing 154 | NM_001143980 | chr16_1426513-1426514_AC_ | 1420_1421delGT | Deletion | Frameshift | CGGAGNCACTC | 16% |
| ST-7 | CCDC83 | coiled-coil domain containing 83 | CCDS8271.1 | chr11_85271352-85271352_A_G | 110E>G | Substitution | Nonsynonymous coding | CCAGGNAAAAA | 48% |
| ST-7 | CDK4 | cyclin-dependent kinase 4 | CCDS8953.1 | chr12_56431144-56431144_G_C | NA | Substitution | Splice site acceptor | TCCTANTTTCA | 41% |
| ST-7 | CDX2 | caudal type homeobox 2 | CCDS9328.1 | chr13_27435263-27435263_C_T | 311V>I | Substitution | Nonsynonymous coding | GGTGANGGTGG | 38% |
| ST-7 | CEP55 | centrosomal protein 55kDa | CCDS7428.1 | chr10_95269516-95269516_C_T | 385R>X | Substitution | Nonsense | AGCTCNGAAAA | 50% |
| ST-7 | CHRND | cholinergic receptor, nicotinic, delta (muscle) | CCDS2494.1 | chr2_233102991-233102991_C_T | 240L>F | Substitution | Nonsynonymous coding | TCTACNTCATC | 42% |
| ST-7 | CIC | capicua transcriptional repressor | CCDS12601.1 | chr19_47483576-47483576_A_G | 208M>V | Substitution | Nonsynonymous coding | CCTTCNTGATC | 78% |
| ST-7 | COL3A1 | collagen, type III, alpha 1 | CCDS2297.1 | chr2_189563113-189563113_G_A | 246G>E | Substitution | Nonsynonymous coding | GCCTGNACCTC | 50% |
| ST-7 | DOCK6 | dedicator of cytokinesis 6 | NM_020812 | chr19_11188765-11188765_C_T | 1240R>H | Substitution | Nonsynonymous coding | CTGCGNGAGAA | 40% |
| ST-7 | DTL | denticleless E3 ubiquitin protein ligase homolog (Drosophila) | CCDS1502.1 | chr1_210283008-210283008_A_ | 103delA | Deletion | Frameshift | AGTGCNGTGGT | 35% |
| ST-7 | EIF4E3 | eukaryotic translation initiation factor 4E family member 3 | NM_001134651 | chr3_71857070-71857070_C_G | 47V>L | Substitution | Nonsynonymous coding | CGGGANCCCGC | 40% |
| ST-7 | EPC2 | enhancer of polycomb homolog 2 (Drosophila) | NM_015630 | chr2_149228093-149228097_AAGAG_ | 587_591delAAGAG | Deletion | Frameshift | AAAACNAGAGA | 29% |
| ST-7 | EYA1 | eyes absent homolog 1 (Drosophila) | CCDS34906.1 | chr8_72346632-72346632_C_T | 294R>Q | Substitution | Nonsynonymous coding | GCAATNGATCA | 47% |
| ST-7 | FMN2 | formin 2 | CCDS31069.1 | chr1_238564096-238564096_A_G | 1713Q>R | Substitution | Nonsynonymous coding | CTCACNGATGA | 32% |
| ST-7 | FUBP1 | far upstream element (FUSE) binding protein 1 | CCDS683.1 | chr1_78201888-78201892_AAATT_ | 1138_1142delAATTT | Deletion | Frameshift | CAATANAATTA | 77% |
| ST-7 | HCAR3 | hydroxycarboxylic acid receptor 3 | NM_006018 | chr12_121766307-121766307_G_A | 311R>C | Substitution | Nonsynonymous coding | GCAGCNGTTGA | 29% |
| ST-7 | hCG_2038200 | lincRNA | ENST00000399196 | chr6_30890004-30890004_G_A | NA | Substitution | NA | TTTTGNCAGCC | 41% |
| ST-7 | HCRT | hypocretin (orexin) neuropeptide precursor | CCDS11421.1 | chr17_37590027-37590029_GCA_ | 22LP>P | Deletion | In-frame deletion | GGGCGNCAGCA | 11% |
| ST-7 | IDH1 | isocitrate dehydrogenase 1 (NADP+), soluble | CCDS2381.1 | chr2_208821357-208821357_C_T | 132R>H | Substitution | Nonsynonymous coding | CATGANGACCT | 33% |
| ST-7 | INSL4 | insulin-like 4 (placenta) | CCDS6459.1 | chr9_5223699-5223699_C_T | 81T>M | Substitution | Nonsynonymous coding | AGGTANGACAT | 88% |
| ST-7 | IPP | intracisternal A particle-promoted polypeptide | CCDS30702.1 | chr1_45957484-45957485_AC_ | 1163_1164delGT | Deletion | Frameshift | CCATANCACAC | 12% |
| ST-7 | IRS2 | insulin receptor substrate 2 | CCDS9510.1 | chr13_109234474-109234474_G_C | 643S>C | Substitution | Nonsynonymous coding | TGCTGNAGCTC | 51% |
| ST-7 | IRS2 | insulin receptor substrate 2 | CCDS9510.1 | chr13_109235684-109235684_G_C | 240L>V | Substitution | Nonsynonymous coding | CTGCANCGTCA | 50% |
| ST-7 | KDM6B | lysine (K)-specific demethylase 6B | CCDS32552.1 | chr17_7692584-7692586_CAC_ | 751VT>V | Deletion | In-frame deletion | GCCGTNACCAC | 13% |
| ST-7 | MAP3K15 | mitogen-activated protein kinase kinase kinase 15 | NM_001001671 | chrX_19354377-19354377_C_T | 391R>H | Substitution | Nonsynonymous coding | CTTTGNGATAC | 50% |
| ST-7 | MAP3K15 | mitogen-activated protein kinase kinase kinase 15 | NM_001001671 | chrX_19443143-19443143_C_G | 53S>T | Substitution | Nonsynonymous coding | CCCCANTCTCG | 33% |
| ST-7 | MAPKAPK2 | mitogen-activated protein kinase-activated protein kinase 2 | CCDS31001.1 | chr1_204925299-204925301_GCC_ | 34QP>Q | Deletion | In-frame deletion | GCGCANCCGCC | 13% |
| ST-7 | NT5M | 5',3'-nucleotidase, mitochondrial | CCDS32581.1 | chr17_17147849-17147849_G_A | 87G>E | Substitution | Nonsynonymous coding | GCCAGNGCTGA | 52% |
| ST-7 | PIK3CA | phosphatidylinositol-4,5-bisphosphate 3-kinase, catalytic subunit alpha | CCDS43171.1 | chr3_180399632-180399634_GAA_ | 109E>- | Deletion | In-frame deletion | ACCGTNAAGAA | 34% |
| ST-7 | PKIB | protein kinase (cAMP-dependent, catalytic) inhibitor beta | CCDS5126.1 | chr6_123088028-123088028_G_A | 76E>K | Substitution | Nonsynonymous coding | AAAATNAAGAA | 16% |
| ST-7 | PKIB | protein kinase (cAMP-dependent, catalytic) inhibitor beta | CCDS5126.1 | chr6_123088029-123088029_A_C | 76E>A | Substitution | Nonsynonymous coding | AAATGNAGAAA | 16% |
| ST-7 | POLA1 | polymerase (DNA directed), alpha 1, catalytic subunit | CCDS14214.1 | chrX_24645781-24645781_C_A | 352Q>K | Substitution | Nonsynonymous coding | AGGATNAGTAC | 29% |
| ST-7 | QKI | QKI, KH domain containing, RNA binding | CCDS5285.1 | chr6_163904546-163904549_GCTG_ | 739_742delGCTG | Deletion | Frameshift | CGCCANCTGGC | 41% |
| ST-7 | RBM47 | RNA binding motif protein 47 | CCDS43223.1 | chr4_40135377-40135377_G_C | 97I>M | Substitution | Nonsynonymous coding | TCGTANATGCG | 30% |
| ST-7 | SALL3 | sal-like 3 (Drosophila) | CCDS12013.1 | chr18_74854714-74854714_G_C | 579A>P | Substitution | Nonsynonymous coding | TGTCGNCCACC | 37% |
| ST-7 | SETMAR | SET domain and mariner transposase fusion gene | NM_006515 | chr3_4320086-4320086_C_G | 11P>R | Substitution | Nonsynonymous coding | ACGGCNTTGTG | 51% |
| ST-7 | SLC35F3 | solute carrier family 35, member F3 | CCDS1600.1 | chr1_232108012-232108012_G_C | 56W>C | Substitution | Nonsynonymous coding | AAATGNTCGCG | 33% |
| ST-7 | SMARCA4 | SWI/SNF related, matrix associated, actin dependent regulator of chromatin, subfamily a, member 4 | CCDS12253.1 | chr19_10984788-10984788_C_T | 813S>L | Substitution | Nonsynonymous coding | TCTCTNGTGAG | 48% |
| ST-7 | SOX11 | SRY (sex determining region Y)-box 11 | CCDS1654.1 | chr2_5750589-5750589_G_C | 95K>N | Substitution | Nonsynonymous coding | GAGAANATCCC | 39% |
| ST-7 | SOX4 | SRY (sex determining region Y)-box 4 | CCDS4547.1 | chr6_21703147-21703148_AA_ | 403_404delAA | Deletion | Frameshift | AGGTGNAGTCC | 87% |
| ST-7 | SPPL2A | signal peptide peptidase like 2A | CCDS10138.1 | chr15_48815602-48815602_C_T | 307R>Q | Substitution | Nonsynonymous coding | CATTTNGAAAC | 16% |
| ST-7 | STAG2 | stromal antigen 2 | CCDS43990.1 | chrX_123023328-123023328_C_G | 521L>V | Substitution | Nonsynonymous coding | GTGCTNTGATT | 43% |
| ST-7 | STS | steroid sulfatase (microsomal), isozyme S | CCDS14127.1 | chrX_7187758-7187758_T_C | 256S>P | Substitution | Nonsynonymous coding | CCATGNCCTAT | 47% |
| ST-7 | TCOF1 | Treacher Collins-Franceschetti syndrome 1 | CCDS43388.1 | chr5_149752495-149752497_GGA_ | 1145VE>V | Deletion | In-frame deletion | CTGGTNGAGGA | 40% |
| ST-7 | TMEM30A | transmembrane protein 30A | CCDS4983.1 | chr6_76022588-76022588_T_C | 346I>V | Substitution | Nonsynonymous coding | ATTAANTACTA | 51% |
| ST-7 | TTC37 | tetratricopeptide repeat domain 37 | CCDS4072.1 | chr5_94831346-94831346_G_A | 1464P>S | Substitution | Nonsynonymous coding | AGATGNCCAGT | 48% |
| ST-7 | XPO1 | exportin 1 (CRM1 homolog, yeast) | CCDS33205.1 | chr2_61569391-61569391_T_A | 681D>V | Substitution | Nonsynonymous coding | CAGGANCTTTC | 44% |
| ST-7 | ZBTB47 | zinc finger and BTB domain containing 47 | NM_145166 | chr3_42675472-42675472_G_T | 207L>F | Substitution | Nonsynonymous coding | AGCTTNTGCAA | 41% |
| ST-7 | ZNF292 | zinc finger protein 292 | NM_015021 | chr6_88027964-88027964_A_G | 2633K>R | Substitution | Nonsynonymous coding | TGATANGACTG | 37% |
| ST-7 | ZZZ3 | zinc finger, ZZ-type containing 3 | CCDS677.1 | chr1_77814411-77814411_A_G | 754V>A | Substitution | Nonsynonymous coding | TATACNCTGGC | 94% |
